# Weather Conditions Drive Dynamic Habitat Selection in a Generalist Predator

**DOI:** 10.1371/journal.pone.0088221

**Published:** 2014-02-06

**Authors:** Peter Sunde, Kasper Thorup, Lars B. Jacobsen, Carsten Rahbek

**Affiliations:** 1 Department of Bioscience, Aarhus University, Rønde, Denmark; 2 Center for Macroecology, Evolution and Climate, Natural History Museum of Denmark, University of Copenhagen, Copenhagen, Denmark; Institute of Ecology, Germany

## Abstract

Despite the dynamic nature of habitat selection, temporal variation as arising from factors such as weather are rarely quantified in species-habitat relationships. We analysed habitat use and selection (use/availability) of foraging, radio-tagged little owls (*Athene noctua*), a nocturnal, year-round resident generalist predator, to see how this varied as a function of weather, season and availability. Use of the two most frequently used land cover types, gardens/buildings and cultivated fields varied more than 3-fold as a simple function of season and weather through linear effects of wind and quadratic effects of temperature. Even when controlling for the temporal context, both land cover types were used more evenly than predicted from variation in availability (functional response in habitat selection). Use of two other land cover categories (pastures and moist areas) increased linearly with temperature and was proportional to their availability. The study shows that habitat selection by generalist foragers may be highly dependent on temporal variables such as weather, probably because such foragers switch between weather dependent feeding opportunities offered by different land cover types. An opportunistic foraging strategy in a landscape with erratically appearing feeding opportunities in different land cover types, may possibly also explain decreasing selection of the two most frequently used land cover types with increasing availability.

## Introduction

Choices made by individuals about when and where to forage may reveal crucial information about a species’ ecological adaptations to its environment and identify favoured habitats for individuals, which contribute to the persistence of the population [1]. To be biologically useful, however, the metrics used to support habitat analyses must be biologically meaningful and used in a relevant behavioural or ecological context [2], [3].

The degree to which animals use habitats in relation to their relative availability (selection) is a widely used index to assess the apparent importance of alternative habitats, i.e. the greater the selection ratio, the higher its assumed “importance” compared to other habitats [4], [5]. Selection ratios have become a cornerstone for the development of increasingly advanced statistical models that incorporate multiple habitat parameters based on comparisons of use vs. availability, so called Resource Selection Functions (RSFs: [6]–[9]). Even though standard RSFs implicitly assume habitats being selected equally across availability, selection ratios may also vary as function of availability (‘functional responses in habitat selection’ [10]). This phenomenon seemingly appears when the analysis is conducted on data covering a mixture of behavioural states where different habitats are selected to fulfil fundamentally different needs, such as activity and rest [11], [12] or different types of resources such as forage and water [13]. It has also been pointed out that functional responses may appear as spurious results from statistical habitat selection analyses that are misaligned to the underlying behavioural processes of choosing habitats from availability, e.g. if the spatio-temporal context within which habitats are differentially selected is incompletely represented in the statistical model [14].

Despite the fact that habitat selection is frequently presented as being constant, habitat selection is a dynamic process that is likely to be influenced by a variety of temporally variable factors such as seasons or weather. As a possible temporally variable external driver on habitat selection by foraging animals, weather is known to influence spatial behaviour and foraging decisions [15]–[19] and affecting diet composition and prey specific predation rates of generalist predators [20], [21]. By failing to quantify the influence of temporal drivers on habitat selection one may therefore risk missing information about the ecological and behavioural basis for the observed species-habitat relationships. Yet, few studies of habitat use or habitat selection incorporate the effects of weather or other temporally dynamic drivers explicitly as explanatory variables (for exceptions see: [22], [23]).

In this study, we analyse the extent to which use and selection of land cover types at home range level by a generalist forager, the little owl (*Athene noctua*), is conditional on temporal (weather, seasons) and spatial variables (distance from nest, habitat availability within the entire home range and in different distance-to-nest intervals). We found that season and weather variables explained more variation in use of the most frequently used land cover categories than did variation in availability. Furthermore, we found that even when adjusted for the temporal context, little owls used certain land cover types more evenly than the variation in availability would predict (‘functional response in habitat selection’). This may suggest that this generalist forager prefers a mixture of alternative foraging habitats rather than the maximum availability of the most selected ones. Our findings emphasize the importance of addressing temporal drivers in habitat selection analyses and illustrate some of the pitfalls of considering habitat selection problems as a static use-availability relation.

## Materials and Methods

### Study Species, Study Area and Study Subjects

The little owl (*Athene noctua,* 170–210 g) is a nocturnally active predatory bird species that is widely distributed in south and central Europe, where it occupies culturally modified habitats such as pastures, farmland and orchards. Compared to similar sized raptors, its diet has a relatively high non-vertebrate proportion such as earthworms (*Lubricidae*) and insects [24]. It locates its prey by walking on the ground or by perching from poles, trees, buildings or other elevated points [25]. Since the mid-20^th^ century, the species has declined drastically in western and central Europe due to agricultural intensification [24]. During the study period (2005–7), the Danish little owl population was estimated at *c*.100 breeding pairs, with an annual decrease of at least 5% due to breeding season food limitation during May to July [26]. Accordingly, little owls were expected to select habitats in order to maximize foraging success, at least during the breeding season.

During April 2005-June 2007, we surveyed 27 adult little owls on14 territories using radio telemetry within a 27×30 km^2^ study area (56°N, 9°E, 0–60 m. a. s. l.) in Denmark. The landscape was intensively cultivated with >80% of the surface being cultivated and/or grazed by livestock. The climate is oceanic and windy with an annual precipitation of 689 mm and annual mean temperature of 7.5°C with January-February (−0.4°C) and July (15.7°C) as the coldest and warmest months (averages for 1961–90 from the Danish Meteorological Institute).

**Figure 1:**
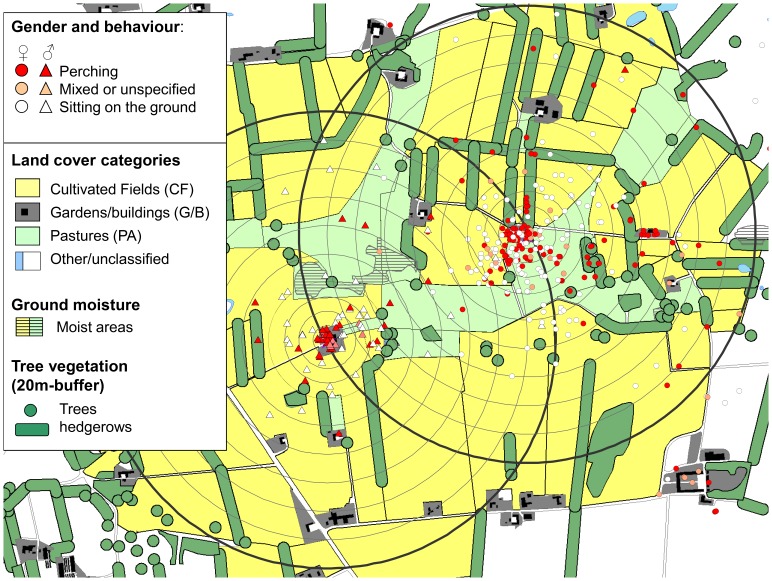
Nocturnal dispersion of four radio-tagged little owls from two pairs. Colour codes indicate whether the owls were perching or were located on the ground, as evident from the strength and echo patterns of the radio signals. Concentric lines 20–800 m from the nest/roosting sites indicate the total area with distance intervals within which habitat use and availability was compared.

The study population comprised monogamous pairs that maintained permanent home ranges throughout the year around a well-defined centre, centred on one or more buildings where they roosted and nested. Home ranges were more or less circular around the nest/roosting site with highly right-skewed activity density distributions (50% of telemetry locations within 125 m, 95% within 800 m [27], Fig. 1). Activity distances from the nest varied with season (shortest May–August, longest January–April) and with temperature (quadratic function: longest distances expressed at temperatures around 5°C [27]). The owls foraged actively from a few metres from the nest site (authors, pers. observations).

### Registration and Selection of Telemetry Observations

Little owls are protected under Danish law. However, ringing and radio-tagging of little owls was carried under license from Copenhagen Bird Ringing Centre with special permit to radio-tag little owls (A-392 personal ringing license to LBJ, and sublicenses A-588 and A-543 to KT and PS). The study was approved by Copenhagen Bird Ringing Centre with permission from the Danish Nature Agency/Danish Ministry for the Environment (J.nr. SN 302-009).

With permission from private owners, we captured little owls in the buildings they used for roosting and nesting. The owls were captured in mist-nets or nest-box traps, following the technical and ethical standards covering capturing, handling and tagging of birds under license in Denmark [28]. The birds were ringed and mounted with backpack VHF radio transmitters (7 g including Teflon harness, TW-4 tags, Biotrack Ltd) with 10–12 months nominal battery life. After completion of the survey, all owls were recaptured and their tags removed. We recorded no cases of accidents or abrasions caused by the tags or the harness, and survival rates of radio-tagged owls were similar to estimates obtained from ring recoveries [29]. Details of the tagged birds and their fates are given in Table A in File S1. Photos of capturing, handling and tagging are available in Figs A-D in File S1.

From public accessible roads and access permission from private land owners, we located the individuals with triangulation from 30 min after sunset to 30 min before sunrise (‘spot observations’). Signals were normally detectable from 0.5–1 km distance with a hand-held directional antenna. If no signal was detectable around a nest location, we systematically searched the surrounding area was for signals in increasing radius until the owl was found. From the strength and ‘echo’ of the signal, we could normally classify whether an owl was located on the ground (weak signal and echo) or perched (stronger signal, less echo). Geographical positions of owls were usually determined with triangulations from 50–100 m distance that were drawn onto maps (1∶10.000) or registered with GPS-navigators. Telemetry fixes with an estimated positioning error of >25 m were removed from the analysis unless they fell well within the limits of a large unit of uniform habitat where reduced spatial precision would not result in misclassification to habitat. To exclude observations of non-foraging owls, we also excluded all telemetry fixes located within 20 m from the nest/roosting site and of vocalising individuals.

Maximum three spot observations from each owl per night were included in the analyses, at least 1 hour apart. Autocorrelation analyses conducted with the Home Range Tools for ArcMap 9.1 [30], showed no significant spatial autocorrelation in locations (Swihart and Slade index <0.6). Each spot observation was assigned wind (Beaufort’s scale), temperature and precipitation measurements.

Seasonal predictors were monthly intervals and (for illustrative purposes) a binary seasonal division made between May–August and September–April. May–August represent the warmest season (mean nocturnal temperature while tracking: 13°C, 98%-observation interval: 5–20°C), where cultivated fields were covered with tall and dense crops and the soil surface was often dry. September–April was the colder season (3°C, 98%-observation interval: −12–14°C) with a wet or humid soil surface and most cultivated fields consisting of bare soil or crop seedling.

### Definition of Land Cover Categories

We created landscape maps (Fig. 1) from existing GIS-layers (TOP10DK database, Danish National Survey and Cadastre) enhanced with our own assessment of the extent of permanent surface categories in August 2006. The land cover types were condensed to four general categories available for all pairs: ‘gardens/buildings’ (G/B), ‘cultivated fields’ (CF, comprised by 61% cereals [barley and wheat, sown in autumn, harvested in July–August], 17% grass cut for hay or silage 2–3 times between May and September, 12% corn [sown in spring, harvested in September-October], 9% dicots [rape, beets and peas]), permanently grazed areas (‘pastures’, PA: 73% cattle grazed, 27% horse grazed) and the remaining surface (‘other’: woods, roads, permanently un-cultivated areas, unclassified open land cover categories on the edges of the home ranges etc.). We categorised all areas within 20 m from trees or hedgerows as being proximate to tree cover (perching opportunities). We categorised ‘ground moisture’ as ‘moist’ or ‘dry’. Because all surfaces categorised to be within G/B were categorised as ‘dry’, the ‘garden/building’ category was excluded from analyses related to ground moisture.

Our analyses of habitat selection (‘the act of using a resource unit if it is encountered’ [9]) considered use relative to availability within the level of the home range (habitat selection on 3^rd^ level following Johnson’s [4] terminology). Hence, the initial selection processes of deciding where to establish home ranges in the landscape as a function of habitat composition (see [31], [32]) are not considered in this paper.

We defined habitat availability relevant for foraging as the area 20–800 m from the nest (‘home range level’), i.e. the area wherein the owls spent 95% of their time. Because habitat composition varied as a function of the distance from the nest (see later), as did the activity density distribution (decreasing density of observations with increasing distance from the nest), we also measured habitat composition within 10 distance-to nest intervals (20–50, 50–100, 100–150, 150–200, 2–300, 3–400, 4–500, 5–600, 6–700 and 7–800 m: ‘distance-from-nest level’) (Fig. 1). Habitat selection analysed on this spatial level was thereby statistically decoupled from the individuals’ initial decision of how far to move from the nest (which was also influenced by seasons and temperatures [27]). Accordingly, habitat selection on ‘distance-from-nest level’ reflected the choice of habitat in relation to availability at a lower hierarchical decision level than when related to the availability of the entire home range. Habitat availability was measured on the basis of a large number regularly distributed ‘availability observations’.

### Analyses

As an initial effort to explore the general pattern of habitat selection, we established habitat selection models that allowed for the effects of multiple habitat features to be incorporated by comparing samples of ‘used’ and ‘available’ habitats within the RSF framework [6]–[8]. The RSFs revealed overall habitat selection patterns in different seasons and identified those habitat features that appeared to be used non-randomly in general or differentially with month. The analysis was conducted as a Generalized Linear Mixed Model (GLIMMIX procedure in SAS 9.2) with a logit-link function and binomially distributed errors [33]. We accounted for individual variation in number of telemetry fixes relative to ‘availability’ observations in the different distance from the nest, by treating subject identity in interaction with log-transformed nest distance as random intercept with degrees of freedoms calculated with the Kenward-Roger approximation [33]. For each subject, we contrasted monthly samples of telemetry fixes with samples of availability fixes, allowing monthly variation in habitat selection to be tested as interaction terms. Computational constraints prevented the analysis to be conducted with any finer temporal resolution than monthly periods.

Because information criteria are unreliable for generalized linear mixed models that contains non-identity links and random effects alongside [34], [35], we evaluated the influences of the explanatory variables on the basis of Type-III F-statistics from analyses of deviance, using p<0.05 as criterion for statistical significance. An unbalanced distribution of available habitat categories among subjects in different distance-to-nest intervals, prevented establishment of models with all possible two-way interactions included. We therefore started with a model including the three land cover definitions (general cover, tree/open, moist/dry) in interactions with month. As tree cover did not have any effect on monthly explicit habitat selection, we reduced the model to consist of general land cover and moisture in interaction with month.

To investigate the temporally explicit variation in habitat selection processes further, we modelled the probability that a given telemetry observation would be located in a given land cover category in question as a function of a set of temporal and/or spatial variables (GLIMMIX procedure with a logit link function and a binomial error term, stating owl identity as a random intercept with degrees of freedoms calculated with the Kenward-Roger approximation). This simple modelling approach had the major advantage that the response variable (the probability that an owl would be located in a given land cover category under a given condition, or the ‘use distribution’, f^U^_(x),_ following Lele et al.’s [9] terminology) was directly interpretable in terms of an activity budget, which could be directly modelled as a combined function of temporal, spatial and life history variables. In this analysis, availability of a given land-cover type (f^A^_[x]_ in Lele et al.’s [9] terminology) was simply measured as the logit-transformed proportion of the number of availability fixes categorised as belonging to it (e.g. if 100 out 500 availability fixes in a section were categorises as G/B, the proportional availability was 100/500 = 0.20 and the logit-transformed availability ln[0.2/{1–0.2}] = −1.39). As follows, the probability that a land cover type would be used as a function of its availability was only modelled for situations where availability was larger than 0 and smaller than 1. Following Mysterud & Ims [10], selection could then be derived as f^U^_(x)_/f^A^_(x)_. As follows, the regression line y = x (f^U^_[x]_ = f^A^_[x]_) in the logit-logit plot suggest random use (habitat is used in the same proportion as it is available), a regression line above y = x that the land cover category is used more than availability would predict and a line below y = x that the habitat is used less than predicted from availability. A slope = 0 suggests that a land cover category is used independently of its availability, a slope >0 that a land cover category is used more the more it is available and a slope = 1 that the land cover category is selected equally across availability. A slope between 0 and 1 indicates that a habitat is increasingly used with increasing availability but at decreasing selection ratio (‘functional response in habitat selection’).

For each class of predictor variables, we found the best combination of predictors by means of log-likelihood tests. After having established predictive models for each class of predictor variables, we combined different class models (e.g. weather effects+seasonal effects) to identify the extent to which different class effects confounded (or in combination improved) the predictive power of the model.

We quantified the predictive power of the different models in explaining variation in habitat use as the maximum rescaled R^2^ and Somer’s D [36]. We regressed the observed binomial outcomes of habitat choice (whether the focal habitat was used) against the predicted probabilities of being used from the GLIMMIX models, using standard logistic regression (LOGISTIC procedure in SAS). The maximum rescaled R^2^ is a measure of the total amount of variation explained by the predictors. Somer’s D is a nonparametric index of a model’s ability to correctly classify the dependent variable, derived as D = 2(AUC –0.5) where AUC is the area under the model’s receiver operation curve. If D = 1, all observations are correctly classified by the model, whereas D = 0 indicates a non-informative model.

Because habitat availability was constant for each subject (owl) throughout the survey, any change in habitat use as a function of temporal variables could by definition also be interpreted as a change in selection. We will therefore in the following sometimes refer to variation in habitat use as a function of temporal variables as change in selection.

## Results

### General Patterns of Use, Availability and Selection

**Figure 2:**
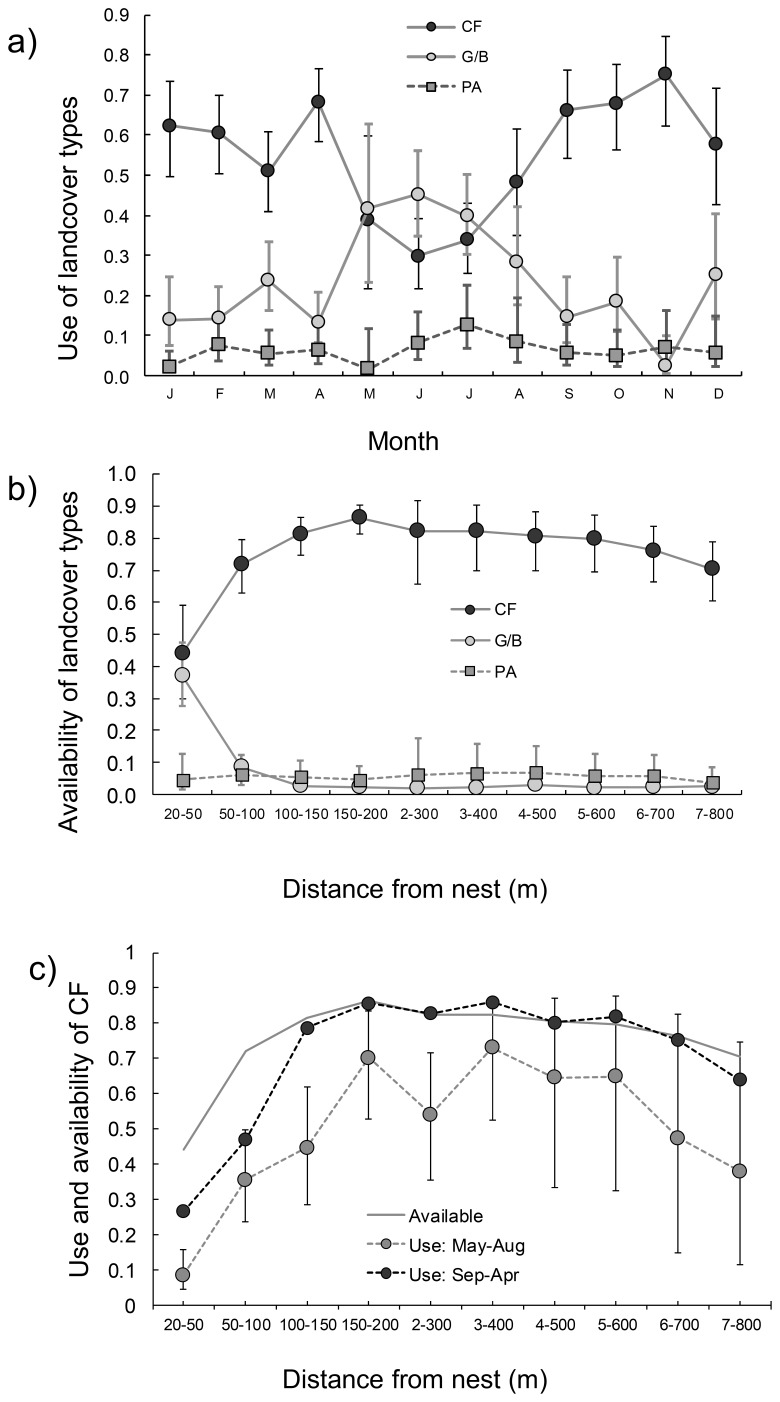
Variation in use and availability of the land cover categories ‘Cultivated fields’ (CF), ‘Garden/buildings’ (G/B) and ‘Pastures’ (PA) of radio-tagged little owls (least square means with 95% confidence limits). (a) Use is divided between months. (b) Availability is divided between distance-to-nest intervals. (c) Availability and seasonal use of cultivated fields is divided between distance-to-nest intervals (confidence errors for use in Sep–Apr are not shown for clarity).

On an annual basis, foraging little owls spent 54% of their time in CF (95% CI: 47–62), as compared to a mean availability of 76% in the home ranges. These mean figures covered a considerable seasonal variation with a peak from September to April and a low in June (Fig. 2a). G/B was the second most frequently used land cover type with an annual mean of 25% (95% CI: 19–32) as compared to 2.9% mean coverage. G/B use peaked in May–July, and dipped in September–April (Fig. 2a). PA represented 7% (95% CI: 4–12) of all use (minor seasonal variation) as opposed to 8% mean availability (Fig.2a). Use as well as availability of G/B decreased and CF increased with increasing distance from the nest (Fig. 2b,c). For the 20 owls with access to ‘moist’ surfaces in their home ranges, mean use were 4% (95% CI: 2–11) as opposed to 6% available.

**Figure 3:**
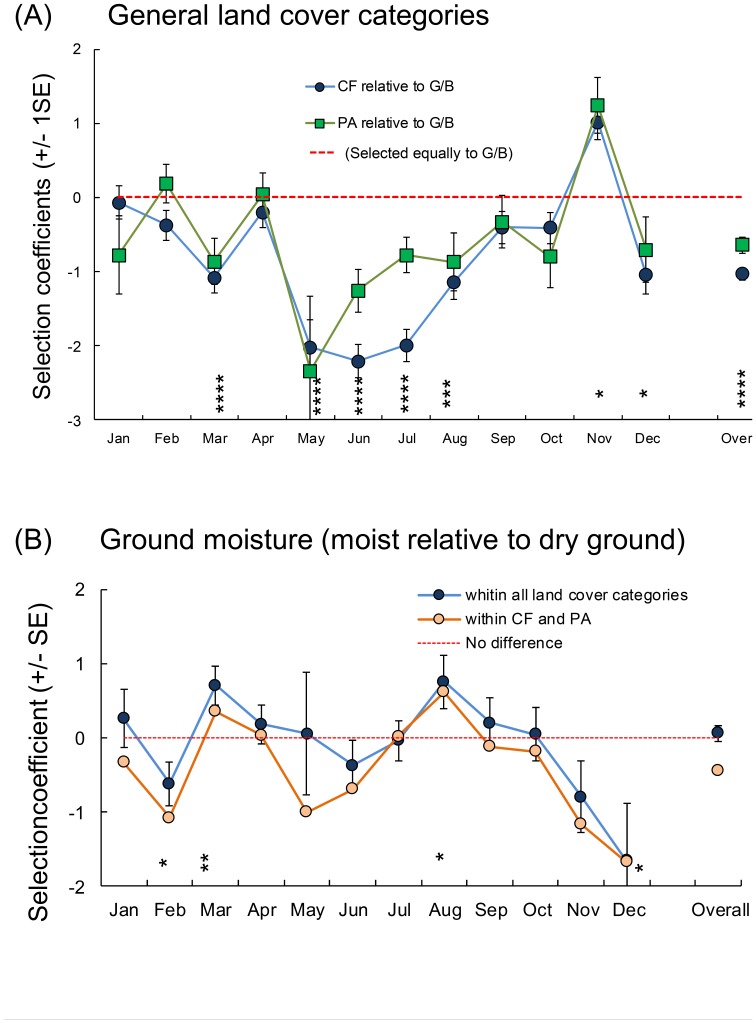
Monthly variation in habitat selection of radio-tagged little owls as predicted from Resource selection functions. A coefficient value of x means that a land cover type is selected exp(x) times more than the reference category. (A) Selection coefficients ‘cultivated fields’ (CF) and ‘pastures’ (PA) relative to ‘gardens/buildings’ (G/B) (Table B in File S2). (B) Selection for ‘ground moisture’ adjusted for month-specific selection of general land cover categories (shown for all four general land cover categories combined [Table B in File S2] and when modelled within CF and PA only [Tables C–E in File S2]). Statistical significances: *: *P*<0.05, **: *P*<0.01, ***: *P*<0.001, ****: *P*<0.0001).

Little owls selected general land cover types (*P*<0.0001) as well as ground moisture categories (*P = *0.008) differently among months (Fig. 3a,b,), but did not select areas in relation to tree vegetation in general (*P* = 0.71) nor in interaction with month (*P* = 0.64) (Tables A–E in File S2).

### Habitat Use as Function of Seasons, Weather and Availability

**Figure 4:**
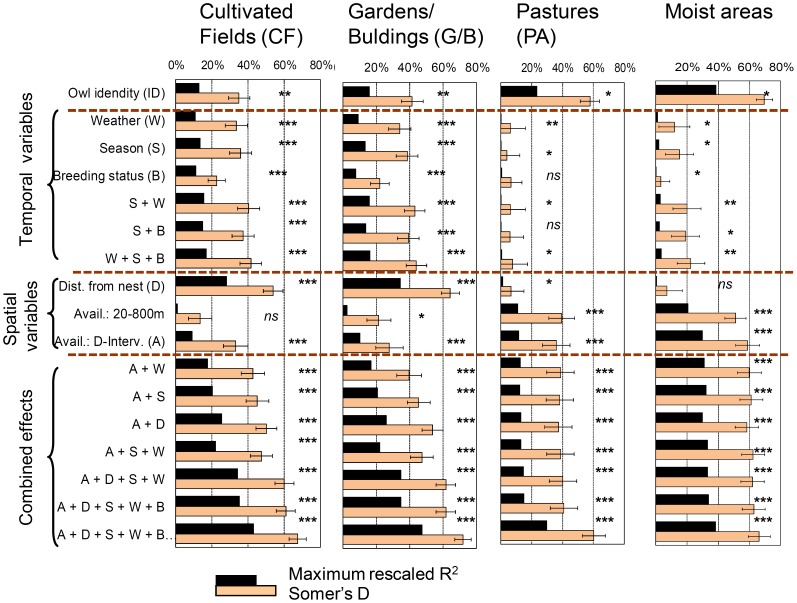
Amount of variation in use of different land cover categories of radio-tagged little owls explained by individual predictor variables and combinations of variables. The maximum rescaled R^2^ expresses the amount of explained variation in terms of reduction of deviance, while Somer’s D (with 95% CIs) expresses a model’s ability to correctly classify whether an owl would be located in a given habitat. Statistical significances; ns: not significant, *: *P*<0.05, **: *P*<0.01, ***: *P*<0.001.

Temporal predictors including weather explained significant amounts of the variation in use of all four focal land cover categories - but the amount of variation explained as well as the specific, influencing variables differed (Fig. 4). Overall, weather and seasons explained most variation in use of CF and G/B and least in PA and ‘moist’ grounds (Fig. 4). Life history variables (sex, breeding phase) explained negligible variation after seasonal effects had been accounted for (Fig. 4, Table A in File S3).

**Figure 5:**
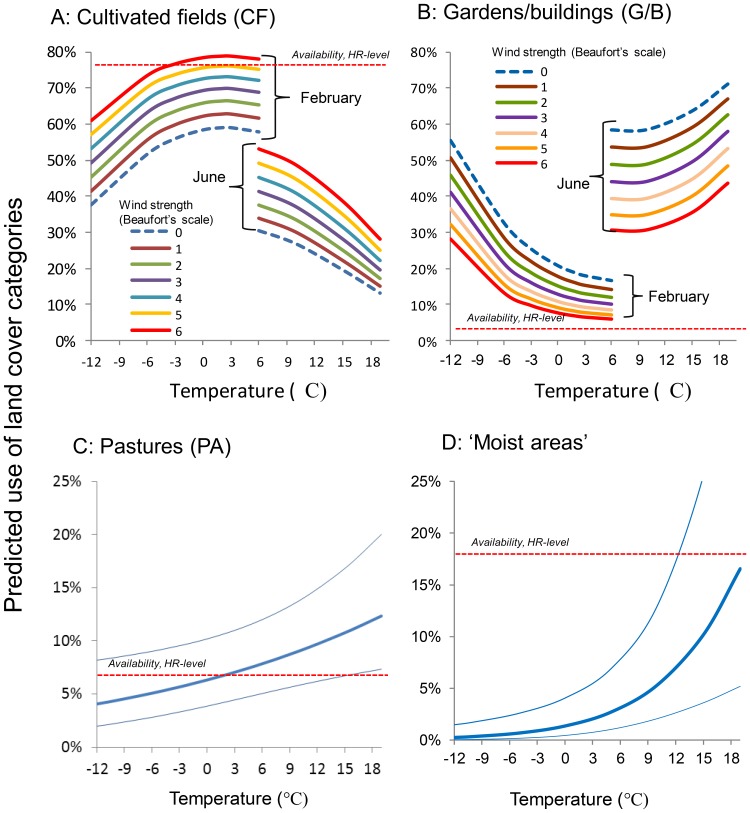
Predicted use of different land cover types of radio-tagged little owls as functions of temperature and wind. The estimates are based on situations where availability at the home range level is equal to the mean for the population (horizontal red dotted lines) and monthly variation is accounted for (see Table B in File S3 for further details). (A) Predicted use of ‘Cultivated Fields’ (CF) as a function of temperature and wind strength shown for February and June. (B) Predicted use of ‘Gardens/buildings’ (G/B) as a function of temperature and wind strength shown for February and June. (C) Predicted use of pastures (PA) as a function of temperature (thin lines show 95% confidence intervals). (D) (C) Predicted use of ‘Moist areas‘ within PA or CF as a function of temperature (thin lines show 95% confidence intervals).

After having accounted for monthly variation and habitat composition at home range level, weather influenced use of CF and G/B through linear effects of wind and quadratic effects of temperature (Fig. 5a,b, Table B in File S3). CF was used more (*P* = 0.0005) and G/B less (*P* = 0.0003) with increasing wind speeds. In relation to temperature, use of CF peaked (adjusted for month: *P* = 0.026), and use of G/B dipped (*P* = 0.0047) at intermediate temperatures, 3–9°C (Fig. 5a,b). In a seasonal context, use of CF correlated positively with temperature in winter and negatively with temperature in summer, whereas the opposite was the case for G/B (Fig. 5a,b). Use of PA and ‘moist areas’ increased with increasing temperatures (*P* = 0.0097 and *P = *0.0003, Fig. 5c,d).

**Figure 6:**
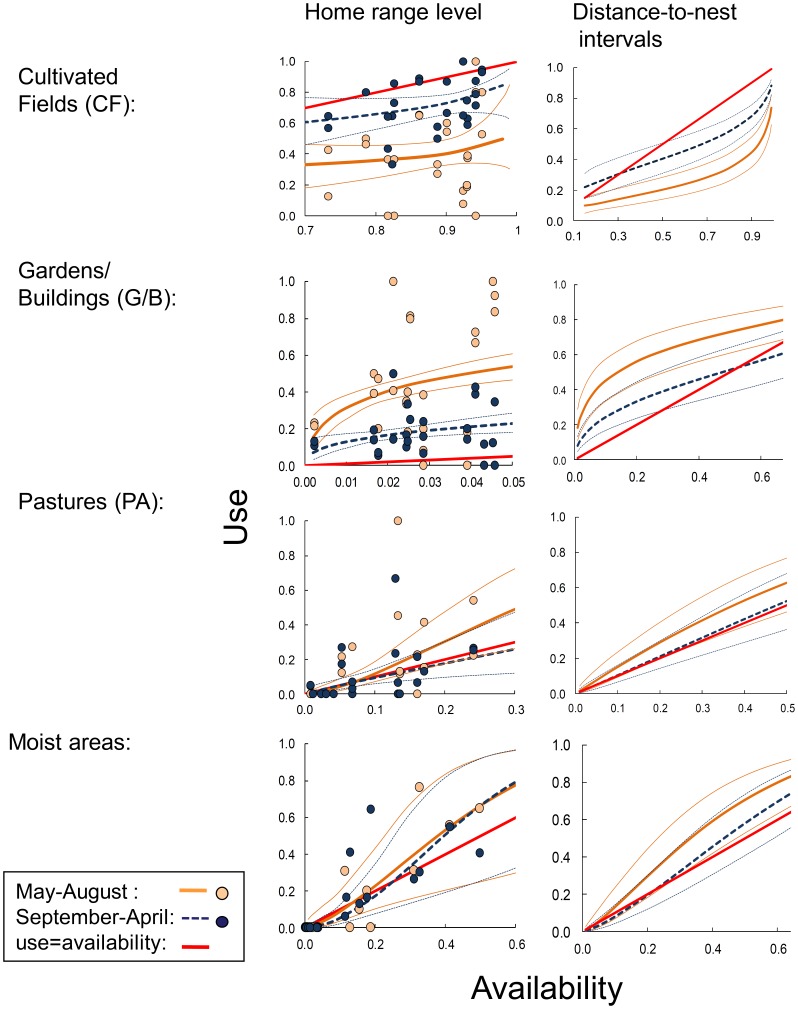
Use of land cover types by radio-tagged little owls in May–August (warm season) and September–April (cold season) plotted/regressed against availability at home range level and within distance-to-nest intervals. At the home range level, each dot represents the proportion of time (telemetry fixes) one owl spent in the land cover category. Regression lines show back-transformed predictions from logit-transformed response variables regressed on logit-transformed proportional cover values (thin lines indicate 95% confidence zones). Predictions above the line y = x suggest that a land cover type is used more than expected by availability; predictions below the line that it is used less than availability would predict. The state space of graphs for distance-to-nest intervals represents the 98%-mid fraction of the availabilities observed.

Use of all four focal land cover categories correlated positively with availability on home range level as well as within distance-to-nest intervals (Table 1, Fig. 6, Tables B–C in File S3), but for CF and G/B, regression slopes of the use-availability functions were significantly <1, showing that these land cover types were used more evenly than predicted from the variation in availability (selected less the more were available: ‘functional response in habitat selection’). For the case of CF, use was seemingly independent of the availability in home range, as indicated by the regression slope not being significantly larger than 0 (Table 1). PA and ‘moist’ areas were used proportional with availability on both spatial scales (Table 1, Fig. 6). For G/B and CF, the distance from the nest explained more variation in use than did nest-distance-specific availability of these land cover types (Fig. 4, Table D in File S3).

**Table 1:** Slopes (*b*) of logistic regression coefficients of the probability that radio-tagged little owls would use focal land cover categories as functions of their logit-transformed (‘availability’) at home range level and at the distance-to-nest interval level.

	Availability at home range level					Availability in distance-to-nest intervals				
	*b*	*SE*(*b*)	*df*	*P*: *b* = 0	*P*: *b* = 1	*b*	*SE*(*b*)	*df*	*P*: *b* = 0	*P*: *b* = 1
Cultivated fields	0.360	0.237	22.1	0.13	0.015	0.585	0.063	1030	<0.0001	<0.0001
Gardens/buildings	0.494	0.210	24.08	0.030	0.026	0.626	0.069	785	<0.0001	<0.0001
Pastures	1.037	0.276	33.29	0.0009	0.39	1.068	0.160	403.7	<0.0001	0.36
Moist ground	1.499	0.392	21.67	0.0012	0.17	1.303	0.160	104	<0.0001	0.07

*P*-values are given for the explicit nil-hypotheses of use being independent of variation in availability (H_0_: *b* = 0) or proportional to availability (H_0_: *b* = 1). The slopes originate from models that also included influence of owl identity (random effect), month and weather variables (Tables B–C in File S3).

## Discussion

This study produced two main results of possible significance for studies of habitat selection patterns in general, and of particular relevance for species with flexible foraging strategies in dynamic environments. First, habitat selection was highly dynamic, and weather factors explained more variation in use of the most frequently used land cover categories, CF and G/B, than did variation in availability. Second, relative to availability measured on two spatial scales, these two land cover categories were used more evenly than variation in availability would predict (functional response in selection). The first result exemplifies that habitat selection, as other behavioural processes is temporally dynamic [22], [23]. The second result adds evidence to the growing understanding that selection ratios may be conditional on availability [2], [3], [37].

### Temporally Dynamic Habitat Selection

While researchers often address seasonally variable habitat selection simply by splitting analyses on seasons [3], fine-scale temporal variation such as weather effects are rarely addressed (for an exception see [23]). The current case may exemplify how incorporation of temporally explicit predictors in the analyses may reveal more information about how an organism is using and selecting habitats in a temporally dynamic world.

In this specific case, seasonal and weather dependent habitat use correlated with variation in diet composition as well as hunting strategies. Hence, in non-frosty periods from September to April, when most foraging took place on CF, the diet consisted almost exclusively of earthworms, whereas in frosty periods, where the G/B was used more, house mice *Mus musculus* (a species strongly associated with buildings), dominated the diet [38]. Since peak use of CF around 0–9°C matches the temperature interval where earthworms are most accessible at the soil surface [39], little owls seemingly concentrated on this easily captured food resource when available, and switched to other prey types when temperatures were too high (summer) or too low (winter) for earthworms being accessible. The switch from CF to G/B between April and May, also matched a structural change of most CF from exposed soils and winter sown crops to dense and tall crops that are less suitable for hunting, since little owls avoid ground vegetation taller than 10 cm [40]. Albeit grazed areas are important foraging habitats for little owls in other populations [25], [40], [41], PA were used much less than CF and G/B throughout the year, and selected less strongly than G/B. This indicate that in the intensively managed Danish farmland, grazed areas did not have a quality to provide attractive alternative foraging opportunities to G/B during the breeding season in May–July when foraging effort peaked [42] and offspring starved [26]. Accordingly, the main dynamic in selection for land cover types appeared to be choosing between foraging in CF and G/B.

Increasing use of CF and decreasing use of G/B with increasing wind speeds may be linked to the efficiency of the predominant hunting strategies used in the two land cover types under different wind regimes. While little owls spent 24% perching (as opposed to being on the ground) in CF, where perch posts are scarce, they perched on 72% of all locations in G/B (Fig. E in File S1). The probability of perching was highly weather dependent (Fig. F in File S1), as it varied as a combined quadratic function of wind strength and temperature (Fig. G in File S1), also after adjusting for seasonal effects and land cover type (Table B in File S1). As the detection ranges of those auditory cues on which nocturnal avian predators rely are generally short [43], and sensitive to wind [44], we find it plausible that at least some of the effect of wind strength on habitat use was ultimately related to decreasing efficiency of perching as foraging strategy in windy weather. We suspect that grazed (PA) and moist areas were increasingly used with increasing temperatures due to temperature related differences in prey availability between these and alternative habitats.

Since the literature is rich in examples of how weather may influence foraging behaviour and diet composition for a variety of reasons [15]–[18], [20], [21], one may speculate whether the scarcity of studies addressing weather effects in analyses of habitat use and habitat selection is due to lack of any such weather variation in most systems, or to not addressing the influence of weather in the analyses. As a result, it is at present difficult to assess the extent to which various temporal drivers influence habitat use decisions in different types of wild animals.

### Relating Use to Availability

This case may illustrate some of the difficulties of interpreting selection ratios [2]. Firstly, the least selected land cover type (CF) was the one most frequently used, contradicting the apparent conclusion that CF were not important foraging habitats. Secondly, habitat composition as well the density of telemetry observations varied as a function of the distance from the home ranges’ activity centres, which obstructed an objective definition of absolute availability. As a further complicating factor for a behavioural interpretation of use-availability relationships, the activity distances were influenced by seasons as well as temperature [27]. The influence of temporal drivers on the owls’ activity distance may also be the reason for nest distance explaining more variation in use of CF and G/B than the nest-distance specific availabilities. Hence, the owls may have decided which land cover type to forage in prior to flying to a given distance-from nest zone with a given habitat composition. Finally, at both the spatial scales at which availability was measured, little owls used the most frequently used land cover types, CF and G/B, more equally than predicted from the variation in availability, i.e. they showed a functional response in habitat selection.

Decreasing selection with increasing availability are known from several large mammals (e.g. roe deer *Capreolus capreolus* [10], red deer *Cervus elaphus* [11], moose *Alces alces* [45], polar bear *Ursus maritimus* [12], African savannah elephant *Loxodonta africana* [13]) and is generally explained as a partitioning of time budgets between competing activities (e.g. foraging and resting) which are associated with different habitats [2], [10], and/or spurious results from statistical selection models that do not match the underlying habitat choice process, e.g. because of unaccounted spatio-temporal effects [14]. From this may follow that animals that only indulge in a single behaviour (e.g. foraging) should show constant selection across availability gradients. This has been found to be the case in tawny owls (*Strix aluco*) that selected woody habitats equally over open habitats along an availability gradient [46]. Increasing selection with increasing availability, as have been reported in raccoon dogs (*Nyctereutes procyonoides)* [47], moose [48], and dispersing passerines in fragmented habitats [49], may possibly reflect habitat switching (changed searching image) beyond a given availability threshold.

The fact that foraging little owls showed decreasing selection of a habitat with increasing availability of that habitat indicates that such functional response in habitat selection may also appear in situations where the same behaviour is expressed and the temporal variation has been accounted for at least to a decent level. A similar pattern was found in hooded crows (*Corvus corone cornix*) that selected forest edges less strongly with increasing availability [50]. As both species can be considered as opportunistic foragers we suggest that the biological reason for the functional responses in habitat selection is an underlying temporal variability in foraging profitability of different habitats too fine-scaled to be incorporated in the temporally explicit use-availability functions. In the present analysis, the land cover categories that could be generalised over all territories represented considerable internal heterogeneity, e.g. different crop types such as winter cereals, grass for cutting and corn provided different seasonal windows of foraging opportunities during the growth season, depending on the seasonal cycle of sowing, growth and harvesting. Harvesting or ploughing could therefore change the profitability of a given patch from nearly unsuitable to highly profitable within hours. Even though this temporal dynamics of land cover types was impossible to quantify in practice, it was obvious from observations of radio-tracked owls taking advantage of erratically appearing feeding possibilities (e.g. foraging extensively on newly ploughed fields) that the owls adjusted their feeding effort in accordance with spatial and temporal variation on scales far too fine grained to be captured by our quantitative measures.

For opportunistic foragers experiencing temporally variable foraging quality of different habitats, functional responses in selection for individual habitat types is perhaps exactly what we should expect as a rule rather than as an exception. As consequence, the most favoured habitat composition for generalist foragers may be a mixture of a range of alternative habitat types that varies in profitability rather than maximum availability of a single “best” one. The way to test this hypothesis is to check for functional responses in habitat selection during foraging with and without controlling for temporal variation.

### Understanding the Behavioural Decisions behind Habitat Selection

The present system exemplifies the complexity of interpreting habitat selection as a behavioural process even in a very simple habitat structure. In the present case, we used a very simple analytical approach based on modelling use as a combined function of temporal and spatial variables. In our case, reducing selection to a choice between binaries could be justified by the simple landscape structure. In more complex landscape structures, more advanced methods to analyse temporally explicit habitat selection relationship may be more suitable [37], although within the RSF framework such tools still remains to be developed (‘existing methods are unable to account for these complications’ [9]). In cases where focal land cover categories of particular interest can be identified, the simple method of modelling use of habitat categories as combined functions of temporal and spatial predictors (including availability) might offer an analytically straightforward supplement for post-hoc investigations upon a more general habitat selection analysis. Use as a response variable conditional on spatio-temporal predictors also has the advantage that it is directly biologically interpretable as a time budget metric, which may be useful if the purpose of the analysis is to achieve mechanistic insight in the underlying reasons for variation habitat selection processes in wild animals.

## Supporting Information

File S1 **Miscellaneous information about the study subjects, radio tagging procedures and variation in perching behaviour that correlated with habitat use.**
(DOCX)

File S2 **Resource Selection Functions for use vs. availability.**
(DOC)

File S3 **Statistical models predicting proportional use of focal land cover categories as functions of temporal and spatial predictors.**
(DOCX)
