# Supplementary material for: Weather Conditions Drive Dynamic Habitat Selection in a Generalist Predator
Source: PLoS One. 2014 Feb 6;9(2):e88221. doi: 10.1371/journal.pone.0088221 (PMC3916403; doi:10.1371/journal.pone.0088221)
Supplement: File S1 — Miscellaneous information about the study subjects, radio tagging procedures and variation in perching behaviour that correlated with habitat use. (DOCX) [file pone.0088221.s001.docx]

**File S1**. Miscellaneous supplementary information.

**Table A**. The radio-tagged little owls: fates, use and availability of land cover categories.

|  |  |  |  |  |  |  | Number of telemetry fixes used in the analysis^1^ | | | | | | | | | | | | | | | Availability, home range level (20-800 m from nest) | | | | | | |
| --- | --- | --- | --- | --- | --- | --- | --- | --- | --- | --- | --- | --- | --- | --- | --- | --- | --- | --- | --- | --- | --- | --- | --- | --- | --- | --- | --- | --- |
| ID | sex | pair | UTM coordinate (nest) | Survey period | | Fate | May-August | | | | | | | | September-April | | | | | | |  |  |  |  |  |  |  |
|  |  |  |  | From | To: |  | CF | | G/B | PA | | Misc | |  | CF | | G/B | PA | | Misc |  | CF | | G/B | PA | | Misc |  |
|  |  |  |  |  |  |  | D | M | D | D | M | D | M | | D | M | D | D | M | D | M | D | M | D | D | M | D | M |
| 1 | F | A | 32 VNJ-2235-0200 | 03-Apr-05 | 20-Jan-07 | Recaptured and tag removed, Feb ’07. | 0 | 3 | 7 | 12 | 0 | 1 | 0 | | 0 | 45 | 13 | 20 | 1 | 4 | 2 | 0.662 | 0.000 | 0.024 | 0.126 | 0.092 | 0.090 | 0.006 |
| 2 | M | A | 32 VNJ-2235-0200 | 03-Apr-05 | 17-Apr-07 | Recaptured and tag removed, July ‘07 | 0 | 17 | 14 | 9 | 0 | 2 | 0 | | 0 | 51 | 8 | 19 | 1 | 2 | 2 | 0.662 | 0.000 | 0.024 | 0.126 | 0.092 | 0.090 | 0.006 |
| 4 | F | C | 32 VNH-3710-9075 | 11-Apr-05 | 27-Sep-05 | Died or dispersed | 0 | 3 | 2 | 0 | 0 | 1 | 0 | | 0 | 6 | 3 | 0 | 0 | 1 | 0 | 0.707 | 0.019 | 0.025 | 0.055 | 0.000 | 0.194 | 0.000 |
| 6 | M | C | 32 VNH-3710-9075 | 16-May-05 | 18-Apr-06 | Recaptured and tag removed, Jan ’07 | 0 | 6 | 2 | 0 | 3 | 2 | 0 | | 0 | 20 | 3 | 0 | 0 | 12 | 0 | 0.707 | 0.019 | 0.025 | 0.055 | 0.000 | 0.194 | 0.000 |
| 7 | M | D | 32 VNH-3580-8811 | 20-Jun-05 | 28-May-06 | Died | 1 | 2 | 3 | 0 | 1 | 1 | 0 | | 8 | 28 | 10 | 0 | 10 | 2 | 0 | 0.730 | 0.178 | 0.017 | 0.004 | 0.047 | 0.021 | 0.004 |
| 8 | F | D | 32 VNH-3580-8811 | 20-Jun-05 | 28-Feb-07 | Died or dispersed. | 2 | 7 | 8 | 0 | 5 | 6 | 0 | | 9 | 41 | 12 | 1 | 22 | 2 | 0 | 0.730 | 0.178 | 0.017 | 0.004 | 0.047 | 0.021 | 0.004 |
| 9 | M | E | 32 VNH-3019-9227 | 27-Sep-05 | 09-Dec-05 | Died | 0 | 0 | 0 | 0 | 0 | 0 | 0 | | 0 | 1 | 0 | 0 | 2 | 1 | 0 | 0.749 | 0.005 | 0.043 | 0.115 | 0.004 | 0.084 | 0.000 |
| 10 | F | E | 32 VNH-3019-9227 | 27-Sep-05 | 28-Feb-06 | Died | 0 | 0 | 0 | 0 | 0 | 0 | 0 | | 0 | 11 | 2 | 0 | 4 | 1 | 0 | 0.749 | 0.005 | 0.043 | 0.115 | 0.004 | 0.084 | 0.000 |
| 11 | F | F | 32 VNH-2906-9229 | 18-Jan-06 | 07-Dec-06 | Recaptured and tag removed, June ’07 | 0 | 9 | 24 | 0 | 0 | 3 | 0 | | 0 | 34 | 23 | 0 | 2 | 6 | 0 | 0.795 | 0.025 | 0.041 | 0.043 | 0.020 | 0.076 | 0.000 |
| 12 | M | G | 32 VNJ-2165-0163 | 19-Jan-06 | 20-Sep-06 | Recaptured and tag removed, July ’07 | 0 | 8 | 7 | 4 | 1 | 0 | 0 | | 0 | 13 | 13 | 1 | 1 | 1 | 0 | 0.777 | 0.000 | 0.021 | 0.076 | 0.076 | 0.040 | 0.009 |
| 13 | F | H | 32 VNJ-2263-1137 | 20-Jan-06 | 16-Oct-06 | Recaptured and tag removed, June ’07. | 0 | 0 | 2 | 0 | 0 | 0 | 0 | | 0 | 21 | 3 | 0 | 0 | 0 | 0 | 0.917 | 0.000 | 0.045 | 0.012 | 0.000 | 0.026 | 0.000 |
| 14 | F | I | 32 VNH-3702-9204 | 12-Feb-06 | 21-Oct-06 | Recaptured and tag removed, July ’07 | 10 | 7 | 4 | 3 | 0 | 0 | 0 | | 11 | 13 | 1 | 0 | 0 | 2 | 0 | 0.512 | 0.255 | 0.002 | 0.000 | 0.121 | 0.028 | 0.081 |
| 15 | M | F | 32 VNH-2906-9229 | 25-Feb-06 | 25-Jul-06 | Died | 0 | 4 | 8 | 0 | 0 | 4 | 0 | | 0 | 14 | 12 | 0 | 2 | 5 | 0 | 0.795 | 0.025 | 0.041 | 0.043 | 0.020 | 0.076 | 0.000 |
| 16 | F | J | 32 VNH-4139-8932 | 05-Mar-06 | 26-Jul-06 | Recaptured and tag removed, July ’07. | 0 | 0 | 0 | 0 | 1 | 0 | 0 | | 0 | 12 | 2 | 0 | 0 | 1 | 0 | 0.788 | 0.000 | 0.039 | 0.127 | 0.000 | 0.045 | 0.001 |
| 17 | M | J | 32 VNH-4139-8932 | 02-Apr-06 | 04-Aug-06 | Recaptured and tag removed, July ’07. | 0 | 4 | 2 | 0 | 5 | 1 | 0 | | 0 | 11 | 3 | 0 | 1 | 1 | 0 | 0.788 | 0.000 | 0.039 | 0.127 | 0.000 | 0.045 | 0.001 |
| 18 | F | K | 32 VNH-2201-6348 | 04-Apr-06 | 27-Jan-07 | Recaptured and tag removed, June ’08 | 0 | 12 | 2 | 0 | 10 | 1 | 0 | | 0 | 12 | 1 | 0 | 2 | 5 | 0 | 0.514 | 0.003 | 0.029 | 0.075 | 0.037 | 0.297 | 0.046 |
| 19 | M | L | 32 vnh-1650-9121 | 06-Apr-06 | 14-Jul-06 | Died | 0 | 1 | 12 | 0 | 0 | 4 | 0 | | 0 | 4 | 0 | 0 | 0 | 0 | 0 | 0.807 | 0.000 | 0.046 | 0.021 | 0.000 | 0.126 | 0.000 |
| 20 | F | L | 32 vnh-1650-9121 | 06-Apr-06 | 10-Dec-06 | Recaptured and tag removed, Jan ’07 | 0 | 5 | 26 | 0 | 0 | 2 | 0 | | 0 | 15 | 8 | 0 | 0 | 3 | 0 | 0.807 | 0.000 | 0.046 | 0.021 | 0.000 | 0.126 | 0.000 |
| 21 | M | K | 32 VNH-2201-6348 | 30-May-06 | 14-Jul-06 | Died | 0 | 6 | 4 | 0 | 2 | 4 | 0 | | 0 | 0 | 0 | 0 | 0 | 0 | 0 | 0.514 | 0.003 | 0.029 | 0.075 | 0.037 | 0.297 | 0.046 |
| 22 | M | I | 32 VNH-3702-9204 | 08-Jun-06 | 17-Apr-07 | Recaptured and tag removed, July ’07. | 7 | 8 | 4 | 3 | 0 | 0 | 0 | | 11 | 8 | 2 | 0 | 0 | 0 | 0 | 0.512 | 0.255 | 0.002 | 0.000 | 0.121 | 0.028 | 0.081 |
| 23 | F | M | 32 VNH-4021-8557 | 19-Jun-06 | 24-Jul-06 | Recaptured and tag removed, July ’07. | 0 | 3 | 12 | 0 | 0 | 3 | 0 | | 0 | 0 | 0 | 0 | 0 | 0 | 0 | 0.643 | 0.160 | 0.025 | 0.022 | 0.014 | 0.123 | 0.013 |
| 24 | M | M | 32 VNH-4021-8557 | 19-Jun-06 | 07-Mar-07 | Recaptured and tag removed, June’07. | 0 | 4 | 12 | 0 | 0 | 0 | 0 | | 0 | 18 | 6 | 0 | 0 | 5 | 0 | 0.643 | 0.160 | 0.025 | 0.022 | 0.014 | 0.123 | 0.013 |
| 25 | F | N | 32 VNH-2476-9706 | 21-Jun-06 | 17-Apr-07 | Recaptured and tag removed, June’07. | 0 | 10 | 7 | 0 | 0 | 2 | 0 | | 0 | 35 | 1 | 0 | 0 | 2 | 0 | 0.914 | 0.000 | 0.018 | 0.029 | 0.000 | 0.029 | 0.010 |
| 26 | M | N | 32 VNH-2476-9706 | 21-Jun-06 | 26-Jan-07 | Recaptured and tag removed, June ’07. | 0 | 12 | 2 | 0 | 0 | 4 | 0 | | 0 | 27 | 2 | 0 | 0 | 0 | 0 | 0.914 | 0.000 | 0.018 | 0.029 | 0.000 | 0.029 | 0.010 |
| 28 | F | G | 32 VNJ-2165-0163 | 13-Nov-06 | 28-Jun-07 | Recaptured and tag removed, June ’07. | 0 | 0 | 9 | 0 | 0 | 0 | 0 | | 0 | 18 | 4 | 4 | 2 | 0 | 0 | 0.777 | 0.000 | 0.021 | 0.076 | 0.076 | 0.040 | 0.009 |
| 29 | F | B | 32 VNH-3858-9365 | 21-Jan-07 | 27-Jun-07 | Recaptured and tag removed, June’08. | 0 | 4 | 1 | 0 | 0 | 0 | 0 | | 6 | 9 | 5 | 1 | 0 | 1 | 0 | 0.354 | 0.188 | 0.029 | 0.000 | 0.005 | 0.403 | 0.022 |
| 30 | M | B | 32 VNH-3858-9365 | 21-Jan-07 | 27-Jun-07 | Recaptured and tag removed, June’08. | 0 | 4 | 0 | 0 | 0 | 1 | 0 | | 10 | 5 | 3 | 1 | 0 | 1 | 0 | 0.354 | 0.188 | 0.029 | 0.000 | 0.005 | 0.403 | 0.022 |

1. Precise telemetry fixes of silent owls 20-800 m from the nest/roosting site. Abbreviations for land cover categories: CF=cultivated field, G/B= gardens/buildings, PA=pastures, MISC=miscellaneous (all other land cover categories), D=dry, M=moist.

**Figure A.** Mistnet for capturing little owls when flying out of building used for roosting.


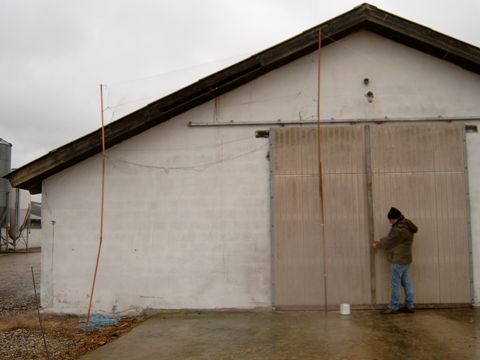


**Figure B.** Little owl captured in mist net.


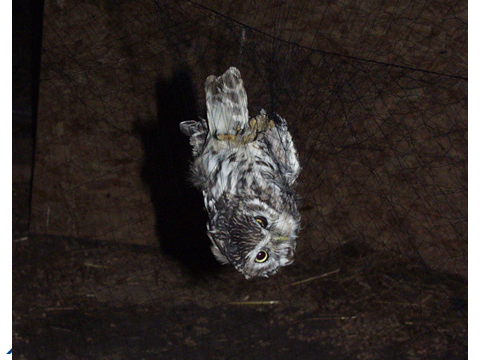


**Figure C.** Mounting of back-pack radio-tag
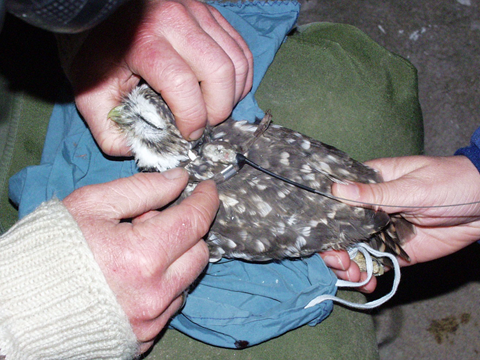


**Figure D.** Radio-tagged little owls ready for being released.


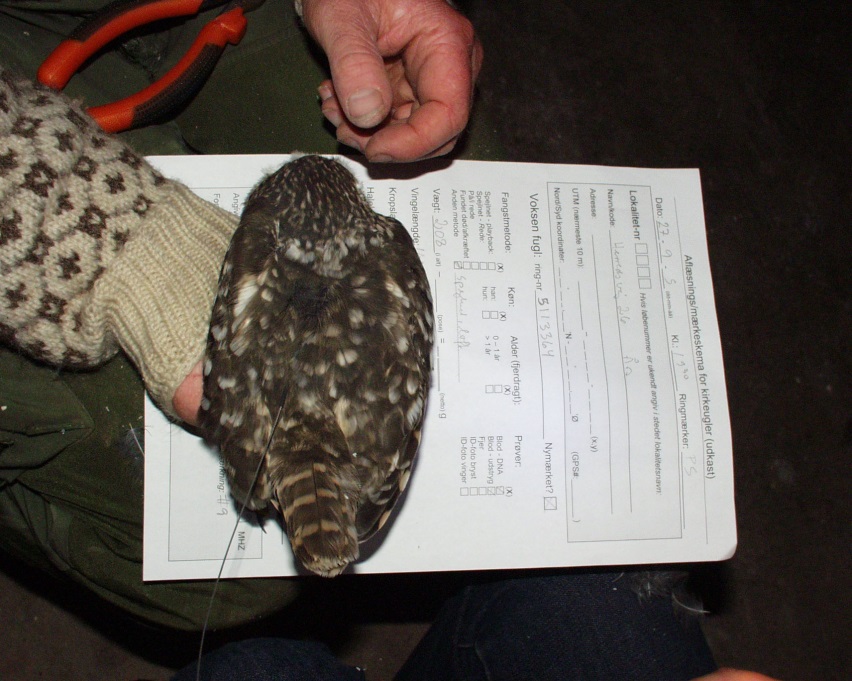

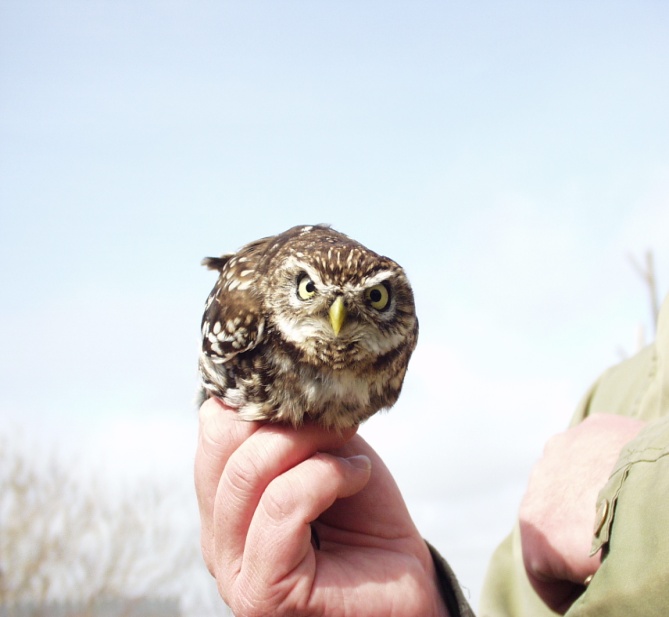


**Figure E.** Estimated probabilities of nocturnally active little owls being categorized as perching (as opposed to being sitting on the ground) from the type of radio signals received (perching owls emitted a clear radio signal from which it was easy to establish a bearing, whereas radio signals from owls sitting on ground were weaker, and associated with a considerable radio ‘echo’. The analytical method of estimating the probability of an owl displaying a specific hunting behaviour was similar to the method estimating the probability of using a given habitat category, as explained in the article.


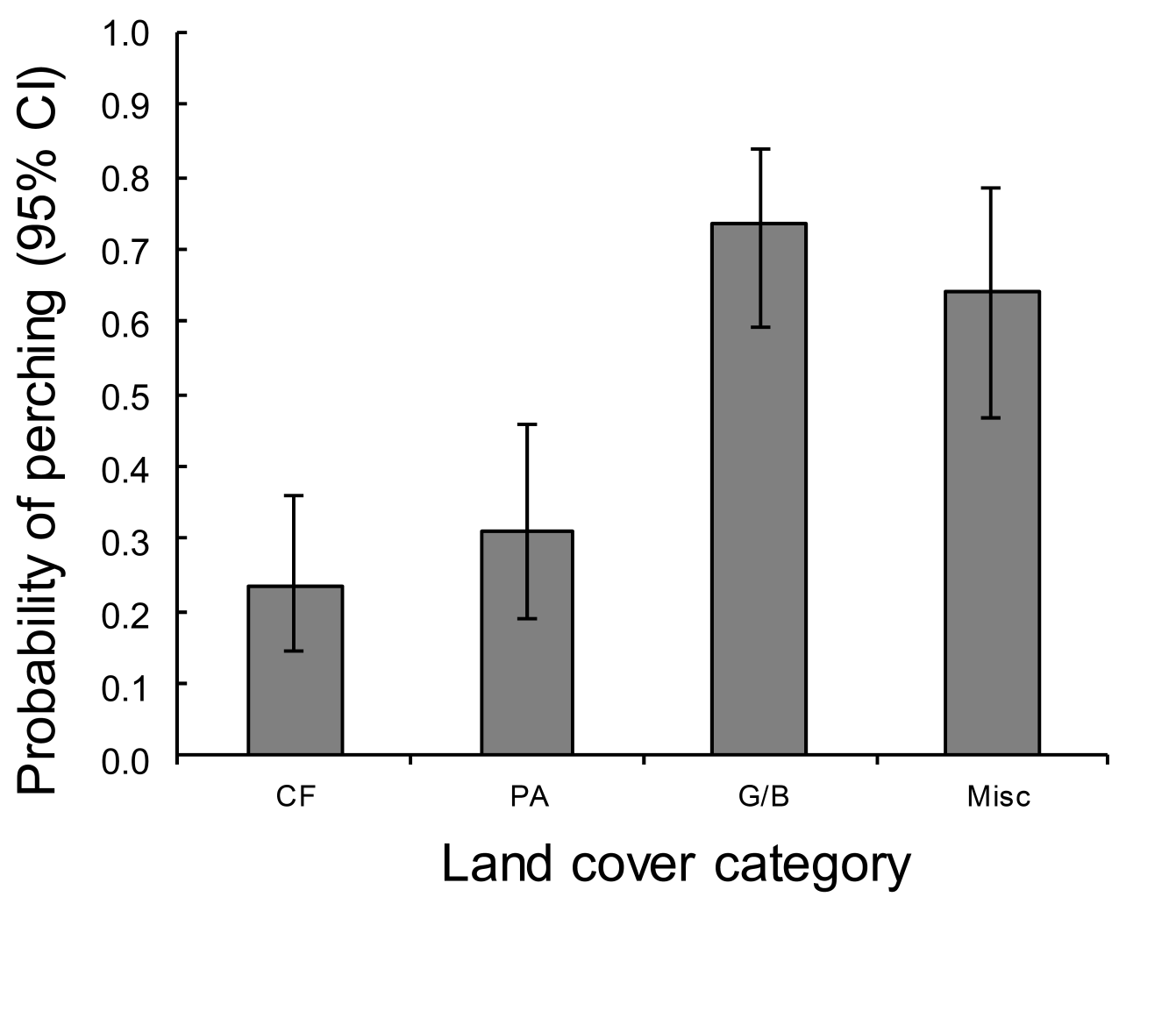


**Figure F.** Predicted probabilities that nocturnally little owls would be perching as a function of variation in temperature and wind strength (generalized linear mixed model with a logit link function and binomially distributed errors, and owl identity treated as a random effect). Equation for predicted functions with SEs in brackets; logit p_perching_ = 0.244 [0.224] – 0.622W [0.126]**** + 0.079 [0.025]W2*** – 0.122[0.023]T**** + 0.010[0.0016]T2**** + 0.0134[0.0068]WT*, where W=wind speed on Beaufort’s scale and T=Temperature in ºC and stars indicate statistical significances: *: p < 0.05, ***: p < 0.001, ****, p < 0.0001.


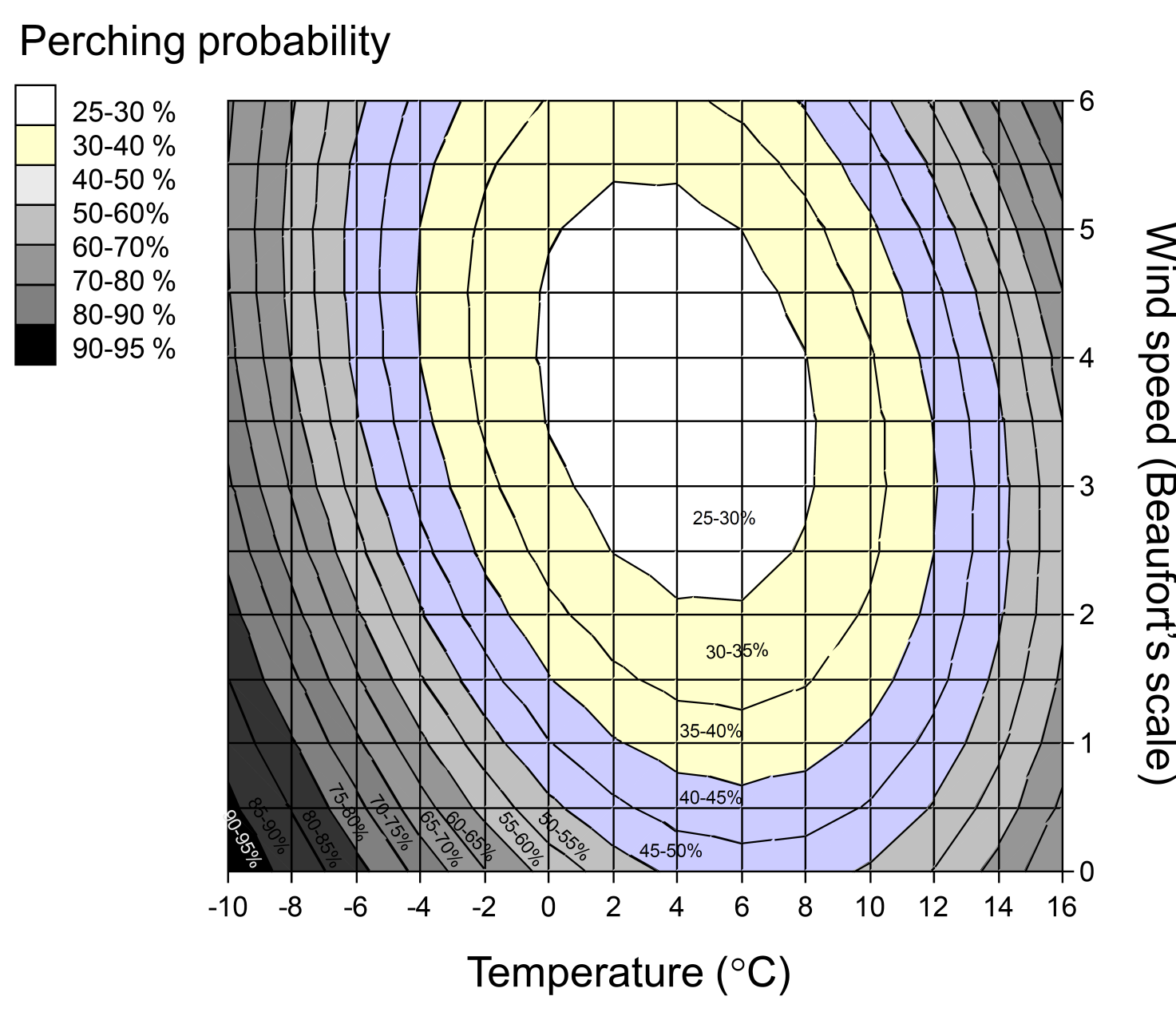


**Figure G.** The amount of variation in hunting strategy (perching vs. non-perching) of little ows, expressed as the maximum rescaled R^2^ and Somer’s D, as explained by different variables and combination of variables. This figure shall be interpreted similarly to Figure 4 in the article, and is based on the same type of analysis (just with hunting strategy as binomially distributed response variable instead of use of a focal land cover type).


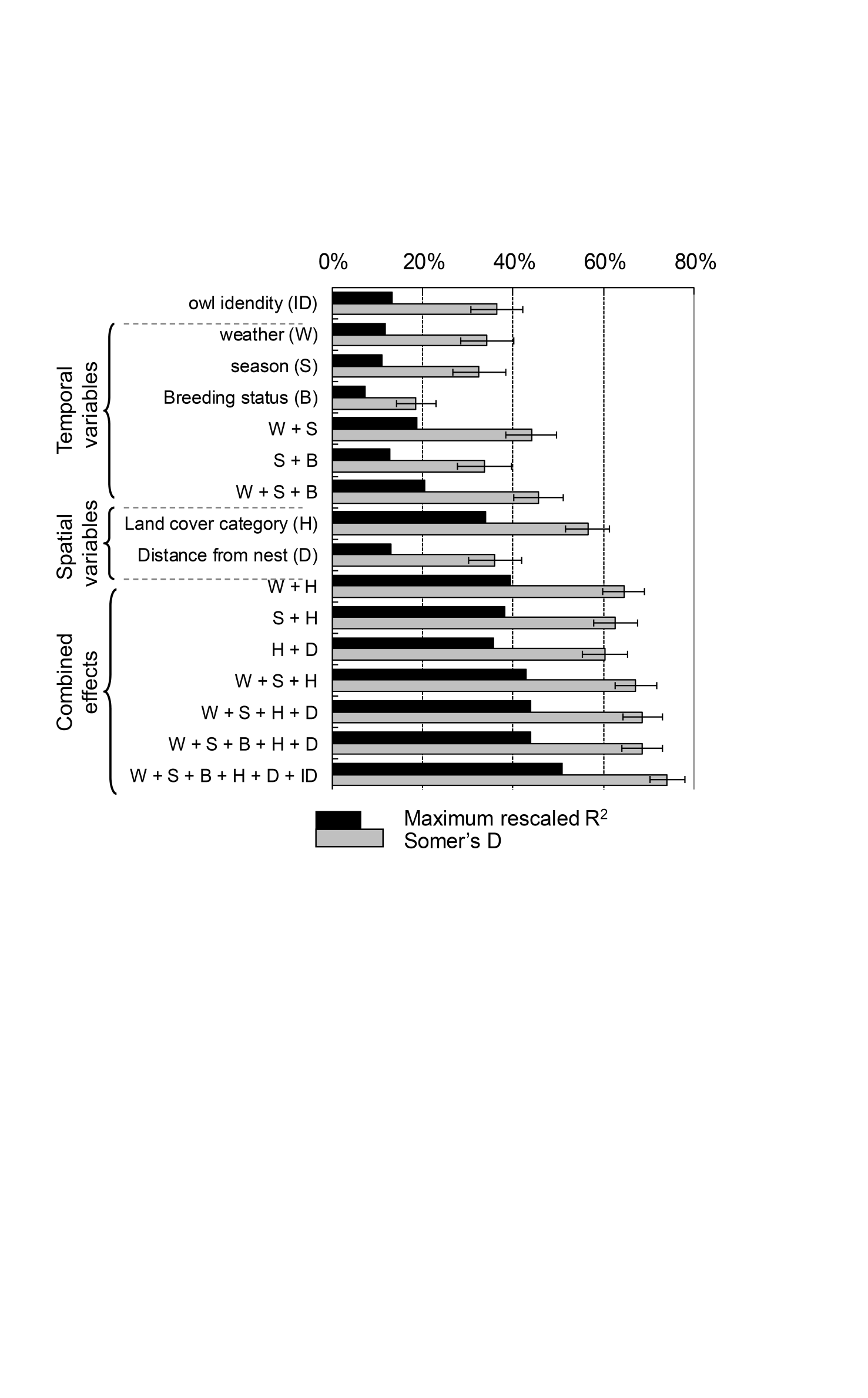


**Table B.** Statistical analysis (generalized linear mixed model with a logit link function, binomially distributed errors, and owl identity treated as a random effect) of variation as of whether radio-tagged little owls were categorized as perching as opposed to sitting at or near the ground when located with telemetry as a function of month, weather variables, land cover categories and distance to the nest.

| **Type III Tests of Fixed Effects** | ***df*** | ***F*** | ***P*** |  |  |
| --- | --- | --- | --- | --- | --- |
| Wind (Beaufort), W | 1,1173 | 13.01 | 0.0003 |  |  |
| W^2^ | 1,1173 | 8.17 | 0.0043 |  |  |
| Temp (°C), T | 1,1173 | 27.14 | <.0001 |  |  |
| T^2^ | 1,1173 | 9.80 | 0.0018 |  |  |
| W*T | 1,1173 | 8.68 | 0.0033 |  |  |
| Month | 11,1173 | 3.68 | <.0001 |  |  |
| land cover category | 3,1173 | 35.22 | <.0001 |  |  |
| log(nest distance, m) | 1,1173 | 28.38 | <.0001 |  |  |
|  |  |  |  |  |  |
| **Equation** | ***b*** | ***SE*** | ***df*** | ***t*** | ***P*** |
| Intercept | 3.865 | 0.672 | 1173 | 5.75 | <.0001 |
| W | -0.519 | 0.144 | 1173 | -3.61 | 0.000 |
| W2 | 0.067 | 0.023 | 1173 | 2.86 | 0.004 |
| T | -0.191 | 0.037 | 1173 | -5.21 | <.0001 |
| T2 | 0.007 | 0.002 | 1173 | 3.13 | 0.002 |
| T*W | 0.024 | 0.008 | 1173 | 2.95 | 0.003 |
| Month=Jan | 0.803 | 0.528 | 1173 | 1.52 | 0.129 |
| Month=Feb | 0.168 | 0.492 | 1173 | 0.34 | 0.733 |
| Month=Mar | 0.725 | 0.521 | 1173 | 1.39 | 0.164 |
| Month=Apr | 0.523 | 0.468 | 1173 | 1.12 | 0.264 |
| Month=May | 1.270 | 0.629 | 1173 | 2.02 | 0.044 |
| Month=Jun | 2.349 | 0.550 | 1173 | 4.27 | <.0001 |
| Month=Jul | 1.359 | 0.550 | 1173 | 2.47 | 0.014 |
| Month=Aug | 0.323 | 0.564 | 1173 | 0.57 | 0.567 |
| Month=Sep | 1.424 | 0.524 | 1173 | 2.72 | 0.007 |
| Month=Oct | 0.894 | 0.491 | 1173 | 1.82 | 0.069 |
| Month=Nov | 0.459 | 0.520 | 1173 | 0.88 | 0.377 |
| Month=Dec | 0.000 | . | . | . | . |
| LCC = CF | -1.832 | 0.215 | 1173 | -8.52 | <.0001 |
| LCC = MISC | -0.104 | 0.304 | 1173 | -0.34 | 0.731 |
| LCC = PA | -1.997 | 0.276 | 1173 | -7.23 | <.0001 |
| LCC = G/B | 0.000 | . | . | . | . |
| log(nest distance, m) | -1.337 | 0.251 | 1173 | -5.33 | <.0001 |
|  |  |  |  |  |  |
| *Owl ID (covariance parameter)* | *0.670* | *0.266* |  |  |  |
