# Supplementary material for: Weather Conditions Drive Dynamic Habitat Selection in a Generalist Predator
Source: PLoS One. 2014 Feb 6;9(2):e88221. doi: 10.1371/journal.pone.0088221 (PMC3916403; doi:10.1371/journal.pone.0088221)
Supplement: File S2 — Resource Selection Functions for use vs. availability. (DOC) [file pone.0088221.s002.doc]

**File S2.** Fixed effects of habitat selection in little owls modelled as monthly explicit Resource Selection Functions.

Statistical significances (Type III effects) and solutions for the fixed effects in the Resource Selection Functions of microhabitat selection. The main effect of month is thus not of biological interest (nuisance variable). The results presented in table A-B are based on a dataset consisting of 1292 selected telemetry observations 20-800 from the nest obtained from 27 owls over 12 months, contrasted with a total sum of 618260 availability observations specific to each owl and month within the same area.

The results presented in Tables C-E are based on a reduced dataset without observations within the general land cover categories ‘gardens/buildings’ (because all areas within this category were dry) and ‘other’ (consisted of a mixture of land cover types which make ecological interpretations difficult), consisting of 681 selected telemetry observations 20-800 from the nest, stratified on 20 owls and 12 months, contrasted with a total of 379.788 availability observations specific to owl and month.

**Table A.** Type III fixed effects of model with effects of adjacency to trees incorporated

| Fixed effect |  | *df* | *F* | *P* |
| --- | --- | --- | --- | --- |
|  |  |  |  |  |
| *Month (M)* |  | *11,* 618000 | *6.68* | *0.29* |
| Land cover category (GLC) |  | 3*,* 618000 | 22.36 | <0.0001 |
| GLC*M |  | 33*,* 618000 | 4.86 | <0.0001 |
| Trees (T) |  | 1*,* 618000 | 0.17 | 0.68 |
| T*M |  | 11, 618000 | 0.84 | 0.70 |
| Soil moisture (SM) |  | 1*,* 618000 | 0.61 | 0.44 |
| SM*M |  | 11*,* 618000 | 2.26 | 0.0097 |

**Table B.** Type III fixed effects of model with effects of adjacency to trees removed (the model on which Fig. 3 in the paper is based).

| Fixed effect |  |  | *F* | *P* |
| --- | --- | --- | --- | --- |
|  |  |  |  |  |
| *Month (M)* | *11, 618000* |  | *9.34* | *<0.0001* |
| Land cover category (LCC) | 3*,* 618000 |  | 22.48 | <0.0001 |
| M*LCC | 33*,* 618000 |  | 4.82 | <0.0001 |
| Soil moisture (SM) | 1*,* 618000 |  | 0.63 | 0.43 |
| M*SM | 11*,* 618000 |  | 2.32 | 0.0078 |

**Table C.** Type III fixed effects of model incorporating interaction between month and soil moisture.

| Fixed effect |  | *df* | *F* | *P* |
| --- | --- | --- | --- | --- |
|  |  |  |  |  |
| *Month (M)* |  | *11, 380000* | 6.58 | <0.0001 |
| General Land cover (GLC) |  | 1, *380000* | 4.36 | 0.037 |
| GLC*M |  | 11, *380000* | 2.21 | 0.012 |
| Soil moisture (SM) |  | 1, *380000* | 7.96 | 0.0048 |
| SM*M |  | 11, *380000* | 2.49 | 0.0040 |
| GLC*SM |  | 1, *380000* | 14.8 | 0.0001 |

**Table D.** Selection coefficients for ‘moist’ as opposed to ‘dry’ ground (year-round).

| Land cover category | *B* | *SE* | *t_380000_* | *P* |
| --- | --- | --- | --- | --- |
| In general | -0.439 | 0.157 | -2.82 | 0.0048 |
| Within cultivated fields (CF) | 0.031 | 0.182 | 0.17 | 0.86 |
| Within pastures (PA) | -0.910 | 0.213 | -4.28 | <0.0001 |

**Table E.** Selection coefficients for ‘moist’ as opposed to ‘dry’ divided on month (Figure 3b in the paper).

|  | *B* | *SE* | *t_380000_* | *P* |
| --- | --- | --- | --- | --- |
| Jan | -0.338 | 0.415 | -0.82 | 0.42 |
| Feb | -1.085 | 0.313 | -3.47 | 0.0005 |
| Mar | 0.351 | 0.269 | 1.3 | 0.19 |
| Apr | 0.032 | 0.276 | 0.12 | 0.91 |
| May | -1.007 | 1.110 | -0.91 | 0.36 |
| Jun | -0.698 | 0.357 | -1.95 | 0.051 |
| Jul | 0.016 | 0.281 | 0.06 | 0.95 |
| Aug | 0.620 | 0.380 | 1.63 | 0.10 |
| Sep | -0.119 | 0.352 | -0.34 | 0.74 |
| Oct | -0.187 | 0.370 | -0.51 | 0.61 |
| Nov | -1.175 | 0.511 | -2.3 | 0.022 |
| Dec | -1.681 | 0.782 | -2.15 | 0.032 |
