# Supplementary material for: Weather Conditions Drive Dynamic Habitat Selection in a Generalist Predator
Source: PLoS One. 2014 Feb 6;9(2):e88221. doi: 10.1371/journal.pone.0088221 (PMC3916403; doi:10.1371/journal.pone.0088221)
Supplement: File S3 — Statistical models predicting proportional use of focal land cover categories as functions of temporal and spatial predictors. (DOCX) [file pone.0088221.s003.docx]

**File S3.** Details on mixed logistic regression models predicting use of focal land cover categories by radio-tagged little owls as a function of temporal and spatial predictors

Details on selected Generalised Linear Mixed Models (logit-link function, binomial errors, owl identity entered as random subject effects, degrees of freedom estimated with the Kenward-Roger approximation in SAS 9.2), predicting the probability that a foraging little owl would be located in different land cover types or ground moisture categories.

General land cover categories were defined as Cultivated Fields (CF), Gardens/buildings (G/B) and Pastures (PA). Separate sets of models estimated the probability that each of these land cover types would be used as opposed to the other two (a fourth land cover category ‘Other’ which included miscellaneous land cover types and unclassified areas which are difficult to interpret biologically, were discarded from the analysis. These analyses were based on 1180 telemetry fixes from 27 little owls, representing 14 home ranges. For analyses of use in relation to availability in distance-to-nest intervals of CF, G/B and PA, 1046, 799 and 656 telemetry fixes were available (observations were discarded if availability for the focal land cover type was 0 or 1).

Soil moisture was categorised as ‘Moist’ ground (as opposed to dry ground: the analysis was restricted to areas covered by CF and PA as G/B did not contain any surfaces with moist ground^[[1]](#footnote-1)^). These analyses were based on up to 845 selected telemetry observations from 14 little owls with access to moist ground within their home range. For analyses of use in relation to availability in distance-to-nest intervals, 543 telemetry fixes were available (observations were discarded if availability was 0 or 1).

*Temporal predictor variables*

- **Month:** Monthly intervals
- **Wind:** wind strength scored on Beaufort’s scale.
- **Rain:** classification of whether the weather was rainy (if the ground was wet by precipitation) or dry.
- **Temp:** Temperature in °C

*Life history variables*

- **Sex:** the gender of the subject.

*Spatial predictor variables*

- **Availability on home range level** (denoted **logit(A_HR_)**) is the logit-transformed proportional cover of the focal land cover type or soil moisture category, 20-800 m from the nest. Hence, if the focal habitat category covers 20% of the home range, logit(A_HR_) = ln(0.2/(1-0.2)) = -1.386.
- **Availability on distance-to-nest level** (denoted **logit(A_dni_)**) is the logit-transformed proportional cover of the focal land cover type or soil moisture category within a given in the distance-to-nest-interval (20-50, 51-100, 101-151, 151-200, 201-300, 301-400, 401-500, 501-600, 601-700 and 701-800 m). Because habitat composition varied systematically as a function of the distance from the nest, and because the little owls’ activity range varied seasonally and as function of temperature, the effect of temporal drivers and availability on habitat choice could not be unequivocally separated statistically if availability was measured at home range level. Selection for a focal land cover or soil moisture category in relation to the availability in that specific nest-distance the subject had decided to move, thus represent the species’ selectivity for that focal habitat independent of its inclination to move away from the nest.
- **Log(nest distance):** log-transformed distance from the nest (m).

**Table A:** Statistical significances (Type III tests of fixed effects) of individual predictor variables of use of the four different focal land cover categories when tested in isolation (‘univariate’) and as partial or additive effects in a complex model comprised by statistically significant variables (p < 0.05: indicated by boldface types) representing effects of weather; season (month); and habitat availability on the distance-to-nest-interval level as tested with Type-III analyses. W + T (italics) represent the combined effect of wind and temperature. Abbreviations for statistical significances; *: *P* < 0.05, **: *P* < 0.01, ***: *P* < 0.001, ****: *P* < 0.0001.

|  | **Cultivated Fields (CF)** | | | |  | **Gardens/buildings (G/B)** | | | |  | **Pastures (PA)** | | | |  | **Moist within CF and PA** | | | |
| --- | --- | --- | --- | --- | --- | --- | --- | --- | --- | --- | --- | --- | --- | --- | --- | --- | --- | --- | --- |
|  | **Univariate** | | **Partial/additive** | |  | **Univariate** | | **Partial/additive** | |  | **Univariate** | | **Partial/additive** | |  | **Univariate** | | **Partial/additive** | |
|  | ***df*** | ***F*** | ***df*** | ***F*** |  | ***df*** | ***F*** | ***df*** | ***F*** |  | ***df*** | ***F*** | ***df*** | ***F*** |  | ***df*** | ***F*** | ***df*** | ***F*** |
| sex | 1,22.4 | 0.22 | 1,20.9 | 0.28 |  | 1,23.4 | 0.15 | 1,22.7 | 0.32 |  | 1,20.4 | 0.52 | 1,15.7 | 0.36 |  | 1,19.0 | 0.06 | 1,5.1 | 0.49 |
| Temp (T) | 2,1177 | 33.8**** | 2,1030 | **3.02*** |  | 2,1177 | 33.8**** | 2,783 | 1.67 |  | 2,1177 | 4.18* | 1,653 | **4.53*** |  | 2,896 | 3.15* | 1,555 | **14.1***** |
| Wind (W) | 1,1163 | 40.1**** | 1,1030 | **8.88**** |  | 2,1175 | 33.8**** | 1,785 | **8.50**** |  | 2,1175 | 1.03 | 1,650 | 0.17 |  | 2,894 | 0.04 | 1,552 | 0.44 |
| *W+T* | *3,1163* | *20.7***** | *3,1030* | ***5.29******* |  | *3,1163* | *29.0***** | *3,782* | ***3.94***** |  | *2,1175* | *4.28** | *2,650* | *2.23* |  |  |  |  |  |
| Rain | 11,178 | 0.00 | 1,1029 | 2.99 |  | 1,1178 | 1.95 | 1,782 | 0.03 |  | 1,1178 | 3.14 | 1,652 | 1.65 |  | 1,897 | 0.36 | 1,554 | 0.81 |
| month | 11,1168 | 9.76**** | 11,1030 | **2.97****** |  | 11,1168 | 8.43**** | 11,785 | **4.32****** |  | 11,1168 | 1.58 | 11,642 | 1.05 |  | 11,887 | 1.69 | 11,555 | **2.61**** |
| Logit(A_HR_) | 1,25.1 | 0.02 | 1,22.1 | 2.30 |  | 1,23.2 | 4.36* | 1,24.1 | 5.56* |  | 1,32.4 | 17.3*** | 1,33.3 | 14.2*** |  | 1,21.4 | 15.3*** | 1,21.7 | 14.5*** |
| Logit(A_dni_) | 1,1030 | 101**** | 11,030 | **85.5****** |  | 1,797 | 100**** | 1,785 | **83.1****** |  | 1,450.3 | 46.3**** | 1,403.7 | **45.1****** | | 1,177 | 51.8**** | 1,97.8 | **67.1****** |
| Log(nest dist.) | 2,1177 | 33.8**** | 2,1028 | 47.8**** |  | 2,1177 | 134**** | 2,783 | 21.1**** |  | 2,1177 | 3.87* | 2,651 | 5.07** |  | 2,896 | 0.10 | 2,553 | 4.47* |

**Table B.** Statistical significance and predictive equations of the least adequate logistic regression models (models comprised by the least number of statistically significant [*P* < 0.05] predictor variables possible) of the probability that focal habitat categories are used by foraging little owls, as functionof weather variables, monthly variation and availability at home range level (logit[A_HR_). T + T^2^ represent the combined, quadratic effect of temperature. Subject identity (owl ID) indicates the random variation between radio-tagged individuals.

| **Type III Tests of Fixed Effects** | | **Cultivated Fields (CF)** | | |  | **Gardens/buildings (G/B)** | | |  | **Pastures (PA)** | | |  | **Moist within CF and PA** | | |
| --- | --- | --- | --- | --- | --- | --- | --- | --- | --- | --- | --- | --- | --- | --- | --- | --- |
|  |  | ***df*** | ***F*** | ***P*** |  | ***df*** | ***F*** | ***P*** |  | ***df*** | ***F*** | ***P*** |  | ***df*** | ***F*** | ***P*** |
| logit(A_HR_) |  | 1,22.1 | 2.30 | 0.14 |  | 1,24.1 | 5.56 | 0.027 |  | 1,33.3 | 14.16 | 0.0007 |  | 1,21.7 | 14.64 | 0.0009 |
| Wind (Beaufort) |  | 1,1162 | 12.16 | 0.0005 |  | 1,1162 | 13.02 | 0.0003 |  |  |  |  |  |  |  |  |
| Temp (°C), T |  | 1,1162 | 0.69 | 0.41 |  | 1,1162 | 7.23 | 0.0073 |  | 1,1177 | 6.71 | 0.0097 |  | 1,885 | 13.22 | 0.0003 |
| T^2^ |  | 1,1162 | 6.45 | 0.011 |  | 1,1162 | 7.78 | 0.0054 |  |  |  |  |  |  |  |  |
| *T + T^2^: combined effect* |  | *2,1162* | *3.65* | *0.026* |  | *2,1162* | *5.38* | *0.0047* |  |  |  |  |  |  |  |  |
| Month |  | 11,1162 | 3.71 | <0.0001 |  | 11,1162 | 4.60 | <0.0001 |  |  |  |  |  | 11,885 | 2.29 | 0.0092 |
|  |  |  |  |  |  |  |  |  |  |  |  |  |  |  |  |  |
| **Equation** |  | ***b*** | ***SE*** | ***df*** |  | ***b*** | ***SE*** | ***df*** |  | ***b*** | ***SE*** | ***df*** |  | ***b*** | ***SE*** | ***df*** |
| Intercept |  | -0.867 | 0.629 | 49.4 |  | 1.974 | 0.911 | 39.8 |  | -0.017 | 0.713 | 23.5 |  | -0.777 | 1.122 | 58.36 |
| logit(A_HR_) |  | 0.360 | 0.237 | 22.1 |  | 0.494 | 0.210 | 24.1 |  | 1.037 | 0.276 | 33.3 |  | 1.499 | 0.392 | 21.67 |
| Wind (Beaufort) |  | 0.159 | 0.046 | 1162 |  | -0.193 | 0.054 | 1162 |  |  |  |  |  |  |  |  |
| Temp (°C) |  | 0.021 | 0.025 | 1162 |  | -0.074 | 0.027 | 1162 |  | 0.039 | 0.015 | 1177 |  | 0.140 | 0.039 | 885 |
| Temp^2^ |  | -0.004 | 0.002 | 1162 |  | 0.005 | 0.002 | 1162 |  |  |  |  |  |  |  |  |
| Month=Jan |  | 1.100 | 0.460 | 1162 |  | -1.216 | 0.516 | 1162 |  |  |  |  |  | 0.742 | 0.895 | 885 |
| Month=Feb |  | 0.790 | 0.404 | 1162 |  | -1.582 | 0.464 | 1162 |  |  |  |  |  | 0.782 | 0.853 | 885 |
| Month=Mar |  | 0.562 | 0.436 | 1162 |  | -1.209 | 0.483 | 1162 |  |  |  |  |  | 2.182 | 0.878 | 885 |
| Month=Apr |  | 0.852 | 0.388 | 1162 |  | -1.236 | 0.444 | 1162 |  |  |  |  |  | 0.839 | 0.822 | 885 |
| Month=May |  | -0.011 | 0.576 | 1162 |  | 0.648 | 0.612 | 1162 |  |  |  |  |  | -0.537 | 1.344 | 885 |
| Month=Jun |  | -0.354 | 0.449 | 1162 |  | 0.373 | 0.482 | 1162 |  |  |  |  |  | -0.717 | 0.911 | 885 |
| Month=Jul |  | -0.103 | 0.473 | 1162 |  | 0.009 | 0.513 | 1162 |  |  |  |  |  | -0.052 | 0.906 | 885 |
| Month=Aug |  | 0.316 | 0.483 | 1162 |  | -0.196 | 0.526 | 1162 |  |  |  |  |  | -0.018 | 0.940 | 885 |
| Month=Sep |  | 1.061 | 0.453 | 1162 |  | -1.059 | 0.507 | 1162 |  |  |  |  |  | -0.309 | 0.891 | 885 |
| Month=Oct |  | 0.933 | 0.422 | 1162 |  | -0.952 | 0.471 | 1162 |  |  |  |  |  | -0.406 | 0.891 | 885 |
| Month=Nov |  | 1.333 | 0.467 | 1162 |  | -2.496 | 0.703 | 1162 |  |  |  |  |  | -0.216 | 0.924 | 885 |
| Month=Dec |  | 0.000 | . | . |  | 0.000 | . | . |  |  |  |  |  | 0.000 |  |  |
|  |  |  |  |  |  |  |  |  |  |  |  |  |  |  |  |  |
| *Owl ID (cov. parameter)* | | *0.44* | *0.17* |  |  | *0.46* | *0.19* |  |  | *0.85* | *0.40* |  |  | *1.78* | *0.89* |  |

**Table C.** Statistical significance and predictive equations of the least adequate logistic regression models (models comprised by the least number of statistically significant [*P* < 0.05] predictor variables possible) of the probability that focal habitat categories are used by foraging little owls, as functions of weather variables, monthly variation and availability at distance to nest level (logit[A_dni_]). T + T^2^ represent the combined, quadratic effect of temperature. Subject identity (owl ID) indicates the random variation between radio-tagged individuals.

| **Type III Tests of Fixed effects** | | **Cultivated Fields (CF)** | | |  | **Gardens/buildings (G/B)** | | |  | **Pastures (PA)** | | |  | **Moist within CF and PA** | | |
| --- | --- | --- | --- | --- | --- | --- | --- | --- | --- | --- | --- | --- | --- | --- | --- | --- |
|  |  | ***df*** | ***F*** | ***P*** |  | ***df*** | ***F*** | ***P*** |  | ***df*** | ***F*** | ***P*** |  | ***df*** | ***F*** | ***P*** |
| logit(A_dni_) |  | 1,1030 | 85.51 | <0.0001 |  | 1,785 | 83.10 | <0.0001 |  | 1,404 | 44.53 | <0.0001 |  | 1,104 | 66.6 | <0.0001 |
| Wind (Beaufort) |  | 1,1030 | 8.88 | 0.0029 |  | 1,785 | 8.50 | 0.0037 |  |  |  |  |  |  |  |  |
| Temp (°C) |  | 1,1030 | 0.03 | 0.86 |  |  |  |  |  | 1,653 | 4.45 | 0.035 |  | 1,520 | 13.5 | 0.0003 |
| Temp^2^ |  | 1,1030 | 5.32 | 0.021 |  |  |  |  |  |  |  |  |  |  |  |  |
| *T + T^2^: combined effect* |  | *2,1030* | *3.01* | *0.050* |  |  |  |  |  |  |  |  |  |  |  |  |
| Month |  | 11,1030 | 2.97 | 0.0007 |  | 11,785 | 4.32 | <0.0001 |  |  |  |  |  | 11,520 | 2.83 | 0.0013 |
|  |  |  |  |  |  |  |  |  |  |  |  |  |  |  |  |  |
| **Equation** |  | ***b*** | ***SE*** | ***df*** |  | ***b*** | ***SE*** | ***df*** |  | ***b*** | ***SE*** | ***df*** |  | ***b*** | ***SE*** | ***df*** |
| Intercept |  | -0.995 | 0.455 | 655 |  | 1.672 | 0.540 | 509 |  | 0.237 | 0.363 | 38.6 |  | -1.303 | 0.848 | 520 |
| logit(A_dni_) |  | 0.585 | 0.063 | 1030 |  | 0.626 | 0.069 | 785 |  | 1.068 | 0.160 | 403.7 |  | 1.303 | 0.160 | 104 |
| Wind (Beaufort) |  | 0.146 | 0.049 | 1030 |  | -0.172 | 0.059 | 785 |  |  |  |  |  |  |  |  |
| Temp (°C) |  | 0.005 | 0.028 | 1030 |  |  |  |  |  | 0.033 | 0.016 | 653 |  | 0.144 | 0.039 | 520 |
| Temp^2^ |  | -0.004 | 0.002 | 1030 |  |  |  |  |  |  |  |  |  |  |  |  |
| Month=Jan |  | 0.663 | 0.500 | 1030 |  | -0.610 | 0.586 | 785 |  |  |  |  |  | 1.170 | 0.930 | 520 |
| Month=Feb |  | 0.494 | 0.442 | 1030 |  | -1.010 | 0.516 | 785 |  |  |  |  |  | 0.931 | 0.898 | 520 |
| Month=Mar |  | 0.035 | 0.483 | 1030 |  | -0.282 | 0.504 | 785 |  |  |  |  |  | 2.875 | 0.917 | 520 |
| Month=Apr |  | 0.609 | 0.420 | 1030 |  | -0.973 | 0.510 | 785 |  |  |  |  |  | 1.337 | 0.859 | 520 |
| Month=May |  | -0.111 | 0.620 | 1030 |  | 0.345 | 0.665 | 785 |  |  |  |  |  | 1.432 | 1.433 | 520 |
| Month=Jun |  | -0.427 | 0.494 | 1030 |  | 0.237 | 0.486 | 785 |  |  |  |  |  | 0.217 | 0.961 | 520 |
| Month=Jul |  | 0.093 | 0.516 | 1030 |  | 0.292 | 0.488 | 785 |  |  |  |  |  | 0.253 | 0.938 | 520 |
| Month=Aug |  | 0.260 | 0.540 | 1030 |  | -0.407 | 0.554 | 785 |  |  |  |  |  | 0.265 | 0.980 | 520 |
| Month=Sep |  | 0.862 | 0.495 | 1030 |  | -0.866 | 0.546 | 785 |  |  |  |  |  | 0.375 | 0.947 | 520 |
| Month=Oct |  | 1.025 | 0.461 | 1030 |  | -1.155 | 0.530 | 785 |  |  |  |  |  | -0.153 | 0.920 | 520 |
| Month=Nov |  | 1.313 | 0.495 | 1030 |  | -2.734 | 0.782 | 785 |  |  |  |  |  | -0.121 | 0.986 | 520 |
| Month=Dec |  | 0.000 | . | . |  | 0.000 | . | . |  |  |  |  |  | 0.000 | . | . |
|  |  |  |  |  |  |  |  |  |  |  |  |  |  |  |  |  |
| *Owl ID (cov. parameter.)* | | *0.68* | *0.26* |  |  | *1.19* | *0.43* |  |  | *0.72* | *0.38* |  |  | *0.006* | *0.068* |  |

**Table D.** Statistical significance and predictive equations of the same variables as presented in Table C, but with the quadratic effect of log-transformed distance to the nest added to the models.

| **Type III Tests of Fixed Effects** | **Cultivated Fields (CF)** | | |  | **Gardens/buildings (G/B)** | | |  | **Pastures (PA)** | | | |  | **Moist within PA and CF** | | |
| --- | --- | --- | --- | --- | --- | --- | --- | --- | --- | --- | --- | --- | --- | --- | --- | --- |
|  | ***df*** | ***F*** | ***P*** |  | ***df*** | ***F*** | ***P*** |  | | ***df*** | ***F*** | ***P*** |  | ***df*** | ***F*** | ***P*** |
| logit(A_dni_) | 1,757 | 18.73 | <0.0001 |  | 1,783 | 6.19 | 0.013 |  | | 1,487 | 41.57 | <0.0001 |  | 1,77.6 | 65.56 | <0.0001 |
| Wind (Beaufort) | 1,1029 | 9.49 | 0.0021 |  | 1,783 | 6.47 | 0.011 |  | |  |  |  |  |  |  |  |
| Temp (°C) | 1,1029 | 0.32 | 0.57 |  |  |  |  |  | | 1,652 | 5.13 | 0.024 |  | 1,519 | 6.87 | 0.0090 |
| Temp^2^ | 1,1029 | 1.61 | 0.21 |  |  |  |  |  | |  |  |  |  |  |  |  |
| T + T^2^ | 2,1029 | 1.52 | 0.22 |  |  |  |  |  | | 4.32 | <0.0001 |  |  |  |  |  |
| Month | 11,1029 | 2.88 | 0.0010 |  |  | 3.52 | <0.0001 |  | |  |  |  |  | 11,519 | 2.9 | 0.0010 |
| log(nest distance) |  | 63.26 | <0.0001 |  | 11,783 | 47.29 | <0.0001 |  | | 1,652 | 3.65 | 0.056 |  | 11,519 | 2.64 | 0.11 |
|  |  |  |  |  |  |  |  |  | |  |  |  |  |  |  |  |
| **Equation** | ***b*** | ***SE*** | ***df*** |  | ***b*** | ***SE*** | ***df*** |  | | ***b*** | ***SE*** | ***df*** |  | ***b*** | ***SE*** | ***df*** |
| Intercept | -4.813 | 0.665 | 1029 |  | 5.896 | 0.829 | 784 |  | | -1.176 | 0.819 | 283.8 |  | 0.244 | 1.294 | 329.4 |
| logit(A_dni_) | 0.309 | 0.071 | 747 |  | 0.219 | 0.088 | 784 |  | | 1.223 | 0.190 | 521.1 |  | 1.281 | 0.161 | 80.32 |
| Wind (Beaufort) | 0.156 | 0.051 | 1029 |  | -0.155 | 0.061 | 784 |  | |  |  |  |  |  |  |  |
| Temp (°C) | -0.016 | 0.029 | 1029 |  |  |  |  |  | | 0.037 | 0.016 | 652 |  | 0.145 | 0.039 | 519 |
| Temp^2^ | -0.002 | 0.002 | 1029 |  |  |  |  |  | |  |  |  |  |  |  |  |
| Month=Jan | 0.298 | 0.519 | 1029 |  | -0.164 | 0.619 | 784 |  | |  |  |  |  | 1.339 | 0.945 | 519 |
| Month=Feb | 0.113 | 0.454 | 1029 |  | -0.599 | 0531 | 784 |  | |  |  |  |  | 1.033 | 0.906 | 519 |
| Month=Mar | -0.391 | 0.500 | 1029 |  | 0.024 | 0.520 | 784 |  | |  |  |  |  | 3.062 | 0.928 | 519 |
| Month=Apr | 0.253 | 0.431 | 1029 |  | -0.508 | 0.525 | 784 |  | |  |  |  |  | 1.487 | 0.870 | 519 |
| Month=May | -0.264 | 0.646 | 1029 |  | 0.688 | 0.709 | 784 |  | |  |  |  |  | 1.667 | 1.444 | 519 |
| Month=Jun | -0.482 | 0.507 | 1029 |  | 0.285 | 0.496 | 784 |  | |  |  |  |  | 0.284 | 0.970 | 519 |
| Month=Jul | -0.327 | 0.536 | 1029 |  | 0.546 | 0.504 | 784 |  | |  |  |  |  | 0.465 | 0.949 | 519 |
| Month=Aug | 0.166 | 0.553 | 1029 |  | -0.363 | 0.560 | 784 |  | |  |  |  |  | 0.336 | 0.983 | 519 |
| Month=Sep | 0.741 | 0.507 | 1029 |  | -0.766 | 0.552 | 784 |  | |  |  |  |  | 0.474 | 0.955 | 519 |
| Month=Oct | 0.814 | 0.471 | 1029 |  | -0.903 | 0.537 | 784 |  | |  |  |  |  | -0.102 | 0.928 | 519 |
| Month=Nov | 1.189 | 0.504 | 1029 |  | -2.552 | 0.776 | 784 |  | |  |  |  |  | -0.112 | 0.992 | 519 |
| Month=Dec | 0.000 | . | . |  | 0.000 | . | . |  | |  |  |  |  | 0.000 | . | . |
| log(nest distance) | 2.130 | 0.268 | 1029 |  | -2.686 | 0.391 | 784 |  | | 0.707 | 0.370 | 652 |  | -0.748 | 0.461 | 519 |
|  |  |  |  |  |  |  |  |  | |  |  |  |  |  |  |  |
| *Owl ID (cov. param.)* | *0.501* | *0.225* |  |  | *0.9116* | *0.3563* |  |  | | *0.691* | *0.355* |  |  | *0.009* | *0.077* |  |

1. More complex model showed that there were no statistically significant variation between PA and CF in the probability that moist ground would be used within one of these two land cover types, given the same availability and temporal context. [↑](#footnote-ref-1)
